# Spatially tunable multiomic sequencing using light-driven combinatorial barcoding of molecules in tissues

**DOI:** 10.1073/pnas.2527896123

**Published:** 2026-05-18

**Authors:** Giorgia Battistoni, Sito Torres-Garcia, Chee Ying Sia, Silvia Corriero, Carla Boquetale, Elena Williams, Anna Cregeen, Karolina Wasilewska, Martina Alini, Nicole Hemmer, IMAXT Cancer Grand Challenge Consortium, Shankar Balasubramanian, Benjamin Czech Nicholson, Gregory J. Hannon, Dario Bressan

**Affiliations:** ^a^Cancer Research UK Cambridge Institute, University of Cambridge, Li Ka Shing Centre, Robinson Way, Cambridge CB2 0RE, United Kingdom; ^b^ Department of Chemistry, University of Cambridge, Lensfield Road, Cambridge CB2 1EW, United Kingdom

**Keywords:** spatial profiling, RNA expression, chromatin accessibility

## Abstract

Mapping the molecular identities and functions of cells within their spatial context is key to understanding the complex interplay within and between tissue neighborhoods. A wide range of methods have recently enabled spatial profiling of cellular anatomical contexts, some offering single-cell resolution. These use different barcoding schemes to encode either the location or the identity of target molecules. However, all these technologies face a trade-off between spatial resolution, depth of profiling, and scalability. Here, we present Barcoding by Activated Linkage of Indexes (BALI), a method that uses light to write combinatorial spatial molecular barcodes directly onto target molecules in situ, enabling multiomic profiling by next generation sequencing. A unique feature of BALI is that the user can define the number, size, shape, and resolution of the spatial locations to be interrogated, with the potential to profile millions of distinct regions with subcellular precision. As a proof of concept, we used BALI to capture the transcriptome, chromatin accessibility, or both simultaneously, from distinct areas of the mouse brain in single tissue sections, demonstrating strong concordance with publicly available datasets. We also developed an integrated instrument that automates combinatorial barcode writing on tissue sections, enabling high-throughput profiling. BALI therefore combines high spatial resolution, high throughput, compatibility with standard histological pipelines, and workflow accessibility to enable tunable spatial multi-omic profiling.

## Significance

Spatial omic technologies profile the molecular make-up of tissues in detail without tissue disruption, producing detailed maps with great potential for basic and applied research. Existing methods share some limitations: an inability to simultaneously profile multiple biomolecule types, high costs and limited scalability to large sample cohorts, and a lack of adaptability across diverse experimental frameworks. We propose a method in which light is used to guide writing of spatial barcodes directly on a wide range of biomolecules, with flexibility in defining the number and size of profiled locations, enabling their parallel high-throughput profiling via DNA sequencing. We believe that this will facilitate large scale application of spatial omics in biology and medicine.

The physical location of a cell and its interaction with its surrounding microenvironment profoundly influence its gene expression, regulatory networks, cell identity, and function. Prime examples are the concerted activation of homeobox genes in response to morphogen gradients in embryonic development (1, 2) or the role of Notch signaling between neighboring cells in controlling embryonic and adult neurogenesis (3) as well as synaptic plasticity (4). Yet, our ability to discover new and more fine-grained examples of spatiotemporal control of biological functions has been limited by the resolution and scope of technologies for deeply profiling the molecular make-up of cells while maintaining spatial information.

The emergence of methods for spatial omics offers the ability to interrogate the identity and function of cells in the context of their local tissue environment. Among many other findings, these technologies contributed to unveiling spatially defined axes of cell differentiation during gut development (5), provided insights into patterns of interactions between maternal and fetal tissues during placental development in early pregnancy (6), and metrics to refine the stratification of cancer patients for improved treatment outcomes (7–9). What was once a technological void is now crowded with solutions that strive to find a balance between the depth at which molecular information is profiled and the resolution of the associated spatial coordinates.

Key to all existing methods is the use of barcodes to either identify target molecules when spatial coordinates are detected by microscopy [e.g., MERFISH (10), SeqFISH (11)/SeqFISH+ (12), StarMAP (13), in situ sequencing (14, 15), FISSEQ (16), ExSeq (17), BARseq (18)] or to encode their spatial coordinates when next generation sequencing is the detection method [e.g., Spatial Transcriptomics (19), Slide-Seq (20)/Slide-SeqV2 (21), Slide-tags (22), HDST (23), Stereo-Seq (24), Pixel-Seq (25), DBiT-seq (26–28), Light-Seq (29)]. The method to assign and decode barcodes defines the characteristics of the technology in question. Optically decoded barcodes offer superior spatial resolution since they preserve tissue histology, yet this comes at the cost of lower measurement depths, as fewer distinct molecules can be profiled simultaneously. On the other hand, nucleic acid barcodes allow an unbiased and broad coverage of gene expression since the target molecules are read-through sequencing, although usually with a coarser spatial resolution due to various technical limitations in the way the barcode is produced and linked to biomolecules. Several techniques adopt a grid-like distribution of spatial barcodes (19–28) that is independent of histological features, posing challenges in accurately capturing the tissue’s structural characteristics. While in some cases the distribution of the barcoded features in the grid can offer subcellular resolution [i.e., HDST (23), Pixel-Seq (25), Stereo-Seq (24)], this comes at the expense of throughput or of the ability to profile large areas of up to many centimeters squared. Furthermore, methods using spatial DNA barcodes do not allow the preselection of regions of interest within the tissue, resulting in sequencing reads being wasted on profiling areas not relevant for the question at hand and further reducing information depth. There are currently few technologies that combine genome- or transcriptome-wide coverage with the fine spatial resolution necessary to profile the molecular phenotype of single cells, and existing approaches do not scale easily to a throughput sufficient to dissect how their interactions within tissue neighborhoods respond to perturbations during development or pathogenesis in large sample cohorts.

The ability to integrate different types of measurements (e.g., mRNA, chromatin, and protein) allows for a more comprehensive characterization of cellular identity and function. Multiomic integration can be achieved either by projecting data collected in parallel from comparable samples onto a reference model (30), or by measurements from the same physical sample (31, 32). A common strategy for accommodating multiomic profiling on a single physical sample is to transform the biochemical diversity of different target biomolecules into a unified chemistry for barcode assignment (33–37). For instance, Share-Seq (32) and DBiT-seq (27, 28) both employ a nucleic acid handle that can be attached to many types of biomolecules to enable subsequent serial ligations to generate a barcode that encodes the cell identity or spatial location, respectively. While these techniques hold promise for capturing spatial multiomic information, they often require custom-made, complex devices or other specialized equipment, limiting their broad accessibility. Moreover, in many cases these workflows do not easily integrate into standard protocols for sample preparation and processing, putting them out of reach for many labs.

To overcome some of the limitations of existing approaches, we developed Barcoding by Activated Linkage of Indexes (BALI), a spatial multiomic sequencing method employing user-defined optical delivery of spatially referenced molecular barcodes directly to target molecules within intact tissues to enable their identification, measurement, and localization by next generation sequencing. To achieve this, a patterned illumination by UV light directs the spatial linkage of barcode subunits by DNA ligase through serial cycles. Each cycle adds short oligonucleotides representing the individual digits of combinatorial barcodes that are appended onto the target molecules. The assignment of spatial barcodes is tunable from millimeters down to micron scale (~5 μm), and it is mostly limited by the technical specifications of the light patterning method. Depending on the complexity in the combinatorial barcode, the number of areas interrogated is similarly scalable, ranging from one up to millions. Our current efficiency of each cycle of ligation is ~86%, which corresponds to an overall ~48% efficiency in encoding a thousand spatial locations. We characterized the efficiency of the ligation-dependent barcode encoding both on solid supports and tissue sections and assessed the resolution of light directed ligation. As a proof of concept, we used BALI to profile the gene expression of two different areas of the embryonic mouse brain and benchmarked it against RNA sequencing after physical dissection by laser capture microdissection (LCM) (38). Additionally, we profiled chromatin accessibility of different areas of the adult mouse brain using a 4-digit barcode, finding strong agreement with publicly available datasets. Last, we combined gene expression profiling and ATAC-seq to obtain a multiomic characterization of two areas of the adult mouse hippocampus. We demonstrated that BALI barcodes can be written with subcellular resolution and high efficiency, theoretically allowing the rapid profiling of thousands of areas. Thus, BALI has the potential to open up new opportunities in spatial biology, offering a histology-aware approach to multiomic profiling of tissues with tunable resolution, throughput, and layering of genomic information.

## Results

### Barcoding by Activated Linkage of Indexes.

**Fig. 1.**
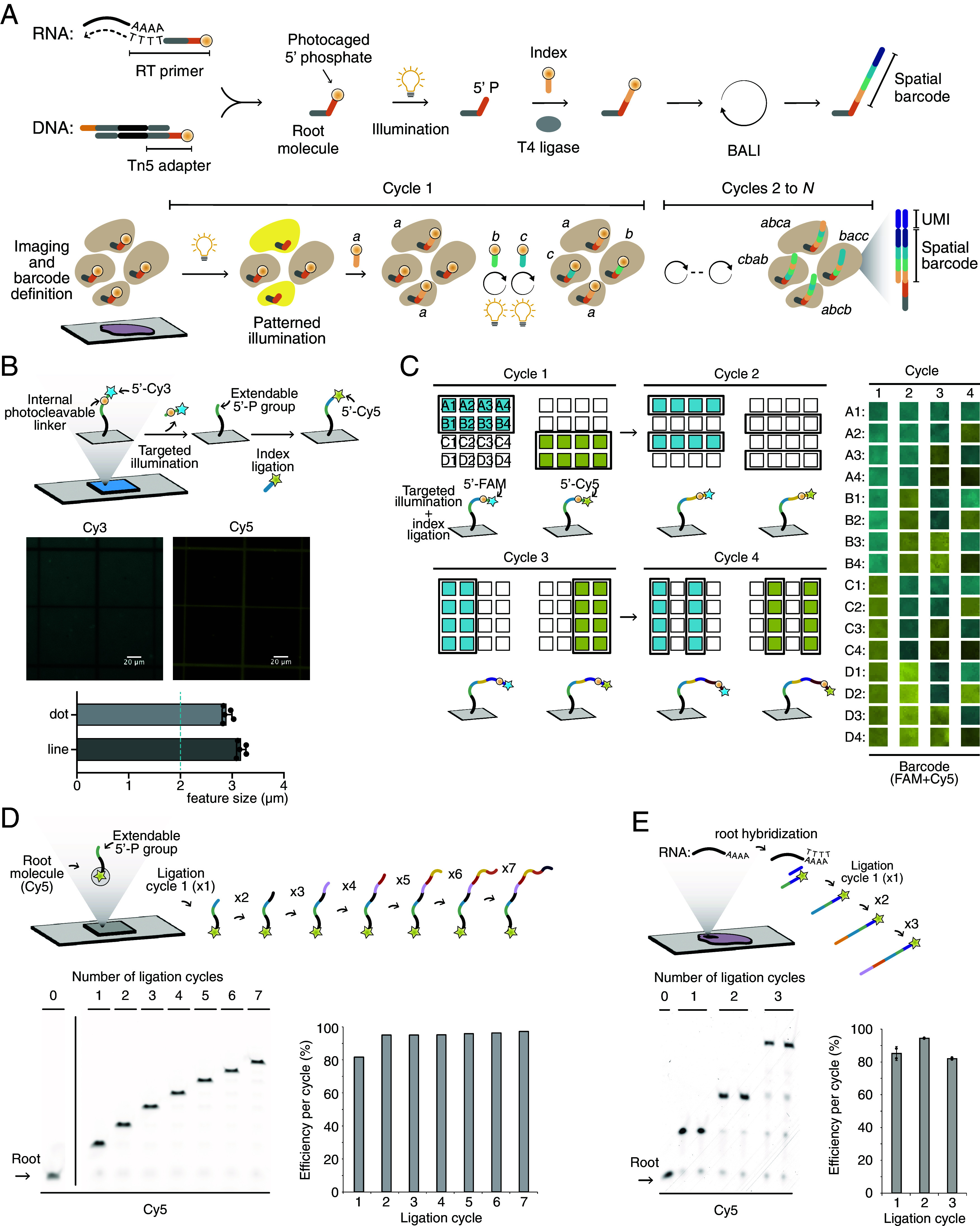
BALI is a technology for spatial-omic profiling that encodes multidigit spatial barcodes to combine high-throughput and high spatial resolution. (*A*) Schematic workflow. Thin tissue sections are placed on glass slides. RT or Tn5 tagmentation are used to couple each target molecule 5’ photocaged ligation root. After imaging, areas of interest are assigned a unique barcode composed by a sequence of DNA “indices,” that are encoded in parallel through rounds of targeted UV illumination and ligation. Each index ligation regenerates an orthogonal caged ligation overhang, allowing cyclic combinatorial barcoding. The barcoded molecules are extracted, amplified, and sequenced. Bioinformatic processing can map each molecule on the genome or transcriptome and in physical space. (*B*) Quantification of BALI barcoding resolution on a solid surface. A schematic of the experimental set up and images of spatially resolved ligation. A slide was covalently functionalized with root oligos blocked by a Cy3 fluorescent photocage. Upon UV illumination, uncaging produced a free 5’P, which was then ligated to a Cy5-fluorescent index. The images show the signal of the anchored oligos (Cy3, *Left*) and ligated cassette (Cy5, *Right*). The bar chart on the *Bottom* shows the measured width (FWHM) across the thin lines of the grid or the central dot (2 μm theoretical size). N = 5, error bars are SD. (*C*) Fluorescent barcodes encoded on a solid surface though four cycles/two indexes. A: schematic of the protocol (*Left*). Root oligos with a photocaged 5’P were attached to a slide as above. Subsequent combinatorial cycles of uncaging and ligation with fluorescent photocaged indices (FAM or Cy5) were used to produce all possible combinations (4 digits ^ 2 variants = 16). Imaging was performed after each cycle and is displayed (panel the *Right*) for all the 16 areas (rows) and cycles (columns). (*D*) Quantification of sequential ligations on Sepharose beads. A schematic of the protocol (*Top*): an oligo featuring a 5’P and 3’Cy5 was annealed to a complementary oligo immobilized on Sepharose beads. The fluorescent oligo was extended through multiple (n = 7) cycles of ligation with DNA indexes. The efficiency of each ligation step was measured by densitometry of the Cy5 signal on a denaturing polyacrylamide gel (*Bottom*). (*E*) Quantification of sequential ligations on thin tissue sections (mouse liver). Schematic of the protocol (*Top*): poly-A mRNA transcripts in thin tissue sections were detected by hybridization with a poly-dT tailed oligonucleotide. This served as molecular docking for a fluorescent ligation root. The root was extended through multiple (n = 3) cycles of in situ ligations. The efficiency of each ligation step was measured by densitometry of the Cy5 signal on a polyacrylamide denaturing gel (*Bottom*). Error bars represent the SD of two replicates. All images are in false colors and color-blind friendly.

At the core of BALI is the use of light to drive sequential ligation of individual oligonucleotide modules to encode combinatorial spatial barcodes directly on intact tissue samples. Initially, tissue sections are placed on conventional histology slides and fixed/permeabilized (Fig. 1*A*). As a first step toward installing the spatial barcodes, a universal ligation root adapter is coupled to target biomolecules in situ. The ligation root is an oligonucleotide with a short (6 nt) single-stranded DNA overhang and a photocaged phosphate at its 5’ end (1-(2-nitro-phenyl)-ethyl based linker) (39). Importantly, this can be installed on target molecules as part of conventional workflows, including reverse transcription and tagmentation, to profile mRNAs and genomic or epigenomic features in a single experiment. Once prepared, the tissue is imaged and segmented into spatial barcode locations of interest, as defined by the user: these can range in size from large tissue regions to subcellular compartments, and in number from few barcodes to millions. Each location is assigned a specific spatial barcode which is then encoded through cycles of illumination and ligation. Upon illumination, the photocleavable linker is released to expose a 5’ phosphate group for ligation to the molecule encoding the subsequent barcode digit with a defined, compatible ligation overhang.

By providing different variant DNA molecules for each digit and using light to control in which region each is installed, it is possible to write complex barcodes combinatorially. The length and complexity of the barcode is determined by the desired number of spatial regions that will be distinguished in each sample. The total number of theoretically possible barcodes is defined as *i^N^*, where *i* represents the number of different identities for each digit of the barcode (also referred to as the barcode’s base), and *N* represents the number of digits in the barcode. For example, a 10-digit barcode with four different values for each digit is sufficient to identify ~ 1 million (4^10^) distinct spatial regions. The base or the number of digits can be further increased to implement codes with error detection and error correction.

During each cycle, all the spatial locations assigned to the same value *i* for the digit *n* to be encoded are illuminated to make their physical spatial barcode molecule extendable (Movie S1). A ligation event appends the index molecule encoding value *i* and regenerates a ligation overhang with a photocleavable cage to the 5’ phosphate, available for writing the following digit. Cycles are repeated to encode all the different values for digit *n* of the spatial barcodes, before moving to following digit and so forth, until the barcode is fully assembled, and all the spatial locations receive a unique DNA identifier with *N* digits. Indexes in the last cycle append a Unique Molecular Identifier (UMI) and an additional primer annealing sequence for PCR amplification. The resulting libraries are prepared and analyzed via sequencing by synthesis with standard 300 cycle reagents (*SI Appendix*, Fig. S1*A*): read-1 (100 bp) is used to map the molecule on either the transcriptome (RNA) or genome (chromatin), whereas read-2 (200 bp) decodes the spatial barcode.

Each digit of the barcode is encoded by a staggered dsDNA molecule, here defined as an “index,” with a specific DNA sequence that is different for each digit value (*SI Appendix*, Fig. S1*B*). The indices are flanked by two orthogonal single strand ligation overhangs on both 5’ ends, each with a different sequence and length (6 or 7 nt). On the strand that extends the ligation root, the ligation overhang includes a photocaged 5’ phosphate.

As a proof of concept, we functionalized a glass slide with a ligation root oligonucleotide carrying a Cy3 fluorophore on its photocaged 5’ phosphate. We illuminated the slide with a grid-like light pattern by a digital micromirror device (DMD) attached to a 405 nm LED. The pattern included sharp lines and dots with a width of 1 pixel, corresponding to a 2 μm theoretical width in the image plane. Illumination resulted in cleavage of the photocage and release of Cy3, as evidenced by the reduction of Cy3 signal in illuminated regions. The removal of Cy3 exposed the 5’ phosphate, which we then used as a substrate for ligation with a short Cy5-labeled dsDNA molecule to mimic the addition of the first digit of a barcode. Both uncaging and ligation were strictly confined to the illuminated areas (Fig. 1*B*), with a calculated level of background uncaging (as measured by the ratio of signals between positive and negative areas) of 1.8%. We determined the ligation resolution by measuring the signal intensity across either lines or dots. The results indicated an actual resolution of ~3 μm, compatible with subcellular measurements. In a separate experiment, we uncaged two separate shapes using the 405 nm diode laser of a commercial confocal microscope at full power. The relative signal of Cy3 and Cy5 was dependent on the amount of light received (*SI Appendix*, Fig. S2), and again we were able to measure a resolution equal or better than 5 μm by measuring a sharp corner of the illumination mask. These results indicate that ligation can be controlled with high spatial resolution in situ.

### Optimal Design of Ligation Overhangs.

In order to optimize the design of our indices, we investigated the dependency of the activity of T4 DNA ligase on the structure of the DNA ends to be adjoined. First, we optimized the length of the overhangs to balance the requirements for strong annealing during ligation while minimizing off-target binding, something often observed with long single-stranded DNA molecules. Across different experimental conditions compatible with BALI, we found that the optimal length for the ligation overhangs was between 6 and 7 nucleotides.

Second, we assessed the sequence dependency of T4 DNA ligase activity. We had observed significant differences in the efficiency of ligation for different overhang sequences, independently of the overhang length and conditions of ligation. Such dependency had been previously described for shorter sticky ends (4 nt) (40), and thus we resolved to expand this analysis to overhangs 6- and 7-nucleotides long. To this end, we screened two individual libraries covering all possible 6-mer and 7-mer overhang sequences to identify those that were able to drive the highest ligation rates in solution (*SI Appendix*, Fig. S3*A*). The screen showed an inverse correlation between the GC content of the ligation overhang and the activity of T4 DNA ligase (*SI Appendix*, Fig. S3*B*). The top 10 hits from each library (GC content > 30%) were individually validated both in solution and in situ for their ability to drive efficient ligation. The validation confirmed the overall ranking of our screening results (*SI Appendix*, Fig. S3 *C* and *D*). Furthermore, we assessed the fidelity of ligation for the top scoring candidates by ensuring no cross-reactivity between different overhangs. This was particularly relevant to avoid the formation of concatemers from the same indices. The top scoring 6-mer and 7-mer sequences were selected as alternating ligation overhangs.

### Efficiency of Barcode Encoding by Iterative Ligation.

Combinatorial barcode encoding by serial ligation is key to the high-throughput nature of BALI. As an initial proof-of-concept of this, we tested whether we could write a 4-digit fluorescent code, in which each digit could have two possible identities (Fig. 1*C* and Movie S2). A 5’ photocaged ligation root was functionalized on a glass slide and subjected to serial cycles of illumination and ligation to extend a DNA barcode. Each alternative index molecule, here defined “A” or “‘B,” carried a different 5’ terminal fluorophore (FAM or Cy3). This allowed us to track the addition (and subsequent uncaging) of index molecules visually. All 16 barcodes could be successfully encoded, as shown in Fig. 1*C*.

The fraction of complete barcodes produced in a BALI experiment depends exponentially on the ligation efficiency of each cycle (*SI Appendix*, Fig. S4*A*). Therefore, high ligation efficiency in every cycle is critical. To measure the individual and cumulative efficiency of barcode writing in our system, we encoded a 7-digit barcode on oligonucleotide-functionalized Sepharose beads (Fig. 1*D*). First, the beads were hybridized to a ligation root consisting of a 5’ phosphate and a 3’ terminal Cy5 fluorophore, which was used to identify the molecules on an acrylamide gel. The efficiency of each ligation cycle was measured by densitometry of the bands at each iteration, while the cumulative efficiency was measured as the product of all the previous cycles. After seven cycles, the cumulative efficiency of ligation was 60.6% with each individual cycle ranging between 81.4% (cycle 1) and >94.8% (cycle 2 through 7). The lower efficiency in cycle 1 was consistent across replicates, which may indicate difficulty in performing ligations in proximity to the bead surface. We also performed a similar experiment to measure the efficiency of barcode ligation in fresh frozen tissue sections from the adult mouse liver following a mild fixation in 0.5% paraformaldehyde (PFA) for 5 min (conditions compatible with both RNA and chromatin accessibility profiling) (Fig. 1*E*). In this case, the fluorescent ligation root was hybridized to a poly-dT oligonucleotide primed on poly-A tails of cellular mRNAs, and we tested three cycles of ligation. The efficiency of ligation was consistently above 80% for all three ligation cycles in each replicate (ranging from 80.6 to 93.8%), with a measured cumulative efficiency of 64.8%. Finally, we investigated whether different fixation conditions or the addition of additives such as bovine serum albumin (BSA) or Triton X-100 to the ligation mix could change ligation efficiency on tissue. Results indicated that ligation efficiency is consistent in the 85 to 90% range with fixation up to 4% PFA for 15 min and addition 4% final BSA (*SI Appendix*, Fig. S5 *A* and *B*).

These efficiencies can be extrapolated to a forecasted efficiency of 48.0% for a 5-digit barcode or 23.1% for a 10-digit barcode, which would label a thousand or million locations, respectively (*SI Appendix*, Fig. S4*B*). BALI is therefore able to encode multidigit spatial barcodes on both solid supports and biological tissue samples to label a large number of areas with high efficiency.

### Spatial RNA Profiling of Mouse Embryonic Brain Regions during Neurogenesis.

One of the fundamental attributes of BALI is its ability to tailor both the quantity and arrangement of the spatial profiling regions according to experimental requirements. The significance of this adaptability is twofold: First, it optimizes sequencing costs by focusing exclusively on relevant locations and, second, it adopts a histology-driven approach to spatial definition, ultimately allowing high-throughput profiling of meaningful locations rather than arbitrary coordinates.

**Fig. 2.**
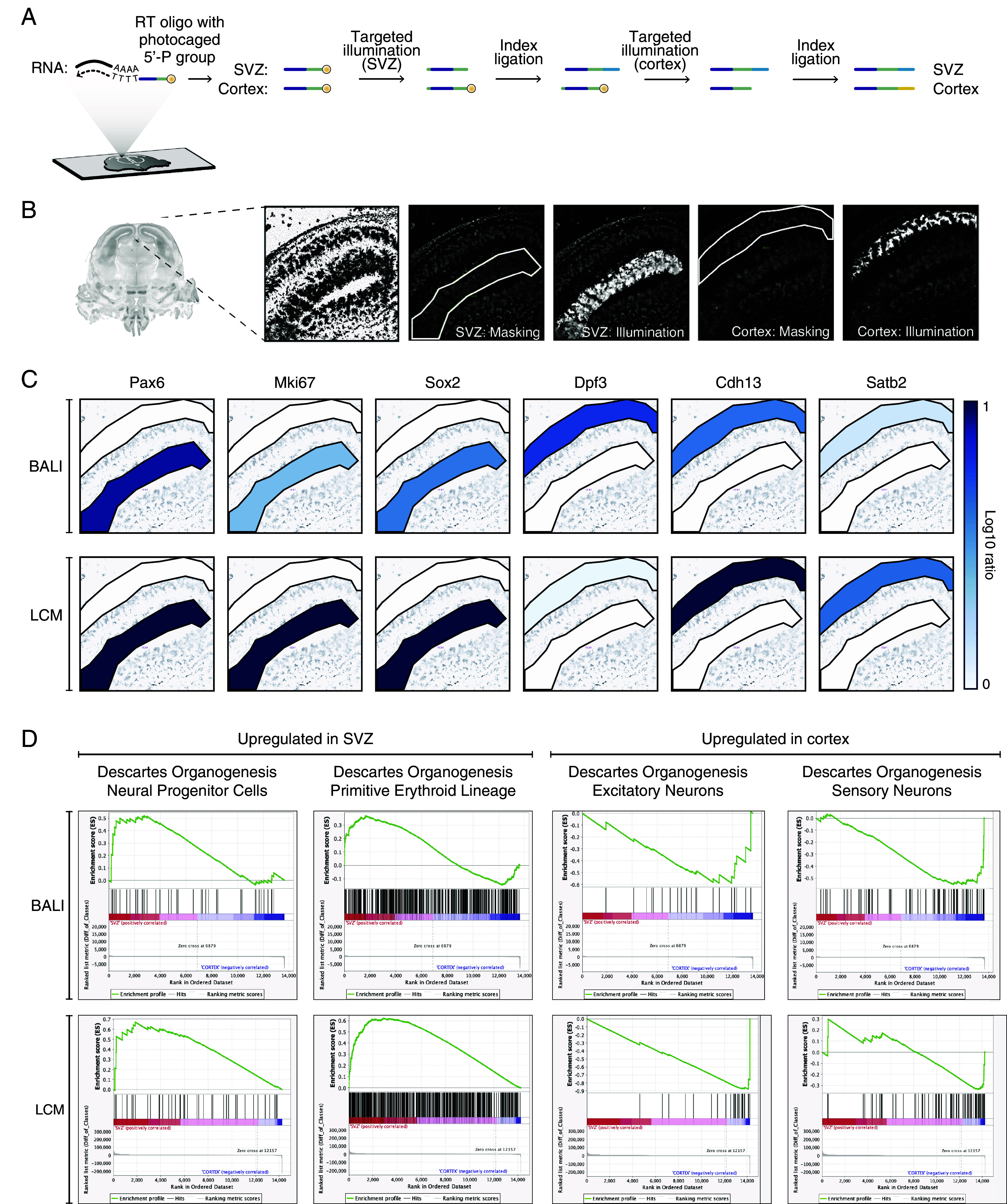
Gene expression profiling of two functional areas of the embryonic mouse brain. (*A*) Schematic workflow. Tissue sections from fresh frozen embryonic day 16.5 mouse brains were placed on microscopy glass slides, fixed, and permeabilized. In situ RT and template switching were performed with an RT including a 5’ terminal overhang (green) ending with a photocaged 5’P (yellow circle). The subventricular zone (SVZ) and cortex of both hemispheres were sequentially UV illuminated to release the photocage and ligated with two different DNA indexes (A:yellow/B:blue). (*B*) Representative microscopy images for the tissue during uncaging. The coronal full section overview is from the Allen Brain Atlas for E16.5 mouse embryonic brains (41). The close-up panels are from our samples (one hemisphere only). The first panel shows the nuclear stain from Draq-5. The following panels show the illumination masks (white outline), and the resulting UV excitation. (*C*) Gene expression of a curated list of markers: expression levels of three marker genes known to be upregulated in the SVZ (*Left*) and cortex (*Right*) are shown in a scale of blue (log10 ratio) over the relevant illumination masks. *Top* and *Bottom* rows represent the expression levels as measured with BALI and LCM (followed by bulk RNA-seq) respectively. (*D*) Gene Set Enrichment Analysis. GSEA outputs for four gene sets from the Descartes gene signature collection (42) in the SVZ (*Left*) or cortex (*Right*) using the expression levels measured with BALI (*Top* row) and LCM (with RNA-seq, *Bottom* row), respectively.

To demonstrate an end-to-end BALI workflow for spatial transcriptomic profiling, we applied the technique to two functionally and anatomically distinct areas of the mouse embryonic brain: the subventricular zone (SVZ) and the developing cortex (Fig. 2 *A* and *B*). In this experiment, the ligation root was introduced during in situ reverse transcription and template switching using a dedicated reverse transcription primer that consisted of a poly-T region at the 3’ end for priming on the poly-A tail of mRNAs, and a photocaged ligation root overhang at the 5’ end. Targeted UV illumination of the SVZ released the photocage from the 5’ phosphate selectively from the cDNA molecules within that region. These molecules were then ligated with an index consisting of a short, staggered dsDNA molecule harboring a defined sequence. The same process was repeated on the cortical region to ligate a second index, producing a simple 1-digit barcode on each region. After ligation of the spatial barcodes, cDNA was purified from the lysed sections and PCR amplified with dedicated primers to generate libraries compatible with Illumina sequencing.

Next, we investigated the expression of a curated panel of marker genes and confirmed the enrichment of cDNAs associated with neural progenitors in the SVZ (e.g., Pax6, Mki67, Sox2) and differentiating neurons in the cortex (e.g., Dpf3, Cdh13, Satb2) (Fig. 2*C*). In addition, Gene Set Enrichment Analysis (GSEA) revealed enrichment of biologically relevant signatures from the Descartes gene signature collection (42), such as “neural progenitor cells” and “primitive erythroid lineage” in the SVZ, and “excitatory neurons” and “sensory neurons” in the developing cortex (Fig. 2*D*).

To benchmark the results obtained with BALI, we performed Laser Capture Microdissection (LCM) followed by bulk RNA-seq on the same brain regions from an adjacent slide. The results from LCM closely matched those obtained with BALI, both in terms of individual marker genes and GSEA outcomes (Fig. 2 *C* and *D*). Therefore, we show that BALI accurately captures the transcriptional profiles of specific spatial regions and yields results consistent with traditional methods such as bulk RNA-seq on physically dissected tissue.

### Spatial Chromatin Accessibility Profiling of the Mouse Adult Brain.

**Fig. 3.**
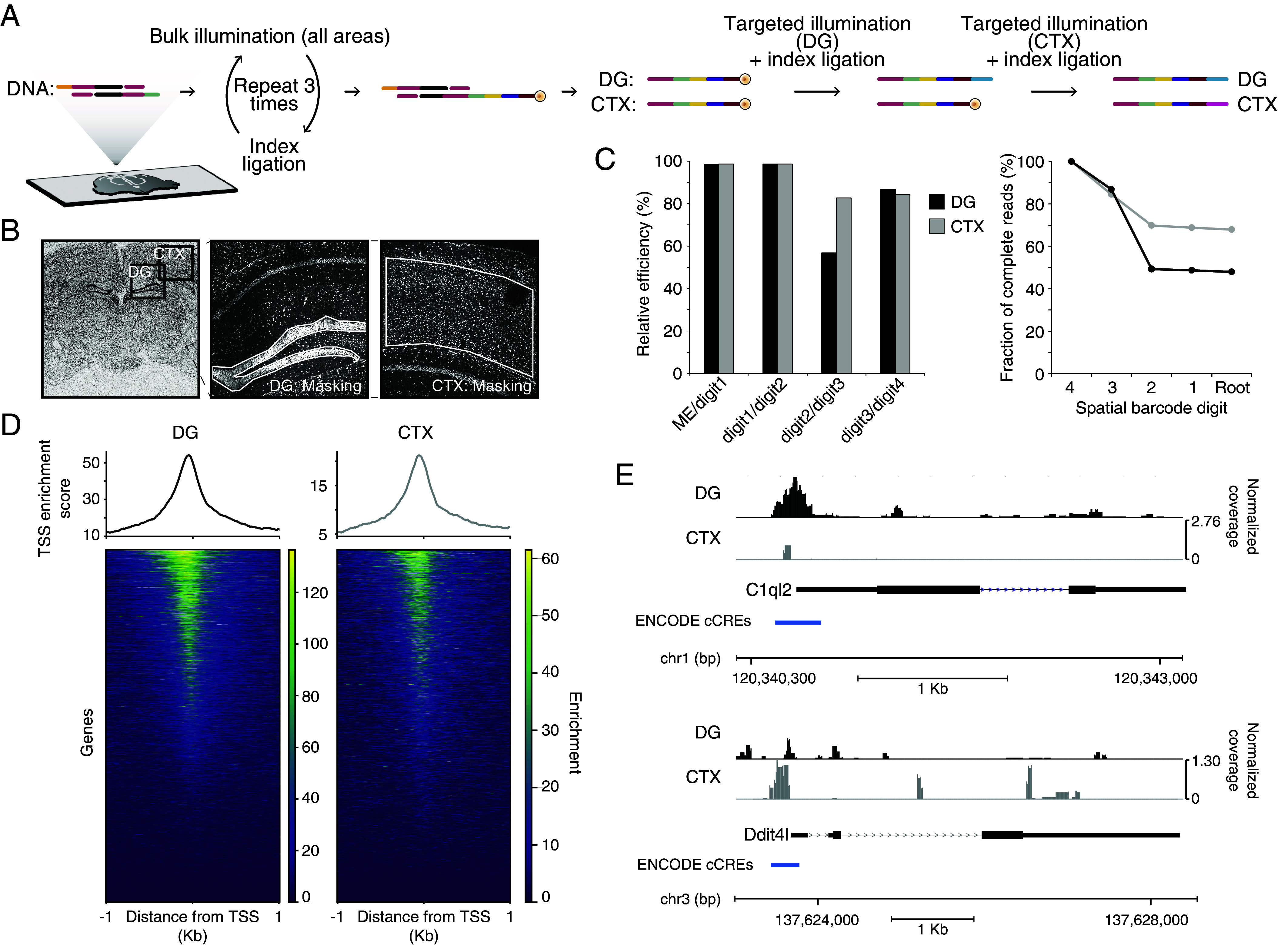
Chromatin accessibility profiling in two areas in the adult mouse brain. (*A*) Schematic workflow. tissue sections from fresh frozen adult mouse brains were placed on glass slides, fixed, and permeabilized. In situ tagmentation with custom adapters was performed targeting accessible chromatin, producing fragments bearing a conventional A-adapter sequence (yellow) (43) and a 5’P overhang (green). Three rounds of ligation and whole-slide illumination extended the root with three different indexes (yellow, purple, magenta) to encode the first, second, and third digits of a 4-digit spatial barcodes, with the last ligation producing a photocaged 5’P. The dentate gyrus (DG) and a region of the adjacent cortex (CTX) were sequentially illuminated and ligated with area specific indices (blue and orange). (*B*) Representative microscopy images of the tissue during uncaging (Draq5 stain). A coronal full section overview of the tissue section, with annotation of the fields of view for uncaging. The close-up panels indicate the illumination masks (white outline). (*C*) Efficiency of ligation for the multidigit barcode for each area. Fractions of full-length barcodes detected are shown as relative percentages for each individual digit (bar plot, *Left*) and cumulative rates (line plot, *Right*). Black refers to the DG, gray to the CTX. (*D*) Enrichment at transcriptional start sites (TSS). The data are shown as cumulative plots (*Top*) and heatmaps for individually ranked TSSs (*Bottom*), for the DG (*Left*), and CTX (*Right*). The graph shown refers to 1 kb upstream and downstream of the TSS. Scales are adjusted for each area. (*E*) Representative coverage plots showing differential chromatin accessibility across two genomic loci. Accessibility for the DG and CTX are shown separately for C1ql2 and Ddit4l. Gene annotations (RefSeq) and promoter annotations [blue, ENCODE cCRE (44)] are shown below.

To expand the portfolio of information accessible through BALI, we applied our method to profile chromatin accessibility within the adult mouse brain. This was achieved by combining in situ Tn5 tagmentation with BALI’s iterative cycles of ligation/illumination on two distinct brain regions: the dentate gyrus and the cortex (Fig. 3 *A* and *B*). In this experiment, the design of the ligation root was slightly modified. A directly accessible 5’ phosphorylated ligation overhang was appended to one of the two adapter oligonucleotides loaded into Tn5, without any photocleavable group present at this step. We introduced this modification to streamline the preprocessing of the tissue, eliminating the need for light-protected conditions during tissue preparation. To thoroughly assess the protocol’s compatibility with multidigit spatial barcodes, a unique 4-digit barcode was assigned to each region. After tagmentation, the first ligation introduced a first shared barcode and an orthogonal ligation 5’ overhang featuring a photocaged 5’ end to all fragments across the tissue. Subsequently, two cycles of bulk illumination and ligation appended the second and third digits of the longer spatial barcode. The fourth digit was then ligated upon targeted illumination to either the dentate gyrus or the cortex.

Next, we lysed the samples to extract the tagmented fragments and generated libraries for sequencing via PCR amplification with dedicated primer sequences. The frequency of spatial reads that included the Mosaic End sequence and a complete barcode was 47.9% and 66.9% for the dentate gyrus and cortex, respectively (Fig. 3*C*). Furthermore, we investigated the rate of occurrence of incomplete barcodes, such as those lacking one or more digits due to failures in intermediate ligations. Given the alternate pattern of ligation overhangs across digits, the most abundant incomplete barcodes were 2-digit barcodes, consisting of digits 3-4 or 1-4, which derived from failures of either the first or second ligation, respectively. Notably, failures in the first ligation were 15 to 30× more frequent than in the second ligation, recapitulating our prior observations on Sepharose beads.

The quality of our ATAC-seq datasets was assessed across a range of different metrics. We obtained a fraction of reads in called peak regions (FRiP score) of 0.48 ± 0.03, a TSS enrichment score of 24.05 ± 5.01 and a percentage of reads mapping in mitochondrial genes of 6.44% ± 1.04%. These values were all within the range of the data standards set by the ENCODE consortium (45) and were comparable to those reported for DBiT-seq in brain tissue (46). The enrichment of fragments in proximity to transcription start sites (TSS) (Fig. 3*D*) showed a skewed distribution immediately preceding the TSS, consistent with open chromatin in promoter regions. Notably, we could detect differential chromatin accessibility in the dentate gyrus and in the cortex. In particular, the promoter region of C1ql2 has been previously shown to be a marker locus for the dentate gyrus in a single-cell ATAC-seq atlas of manually dissected brain regions (47). Our data show a similar chromatin accessibility profile that is specific for the dentate gyrus (Fig. 3*E*). We also investigated the promoter and enhancer regions of genes that are differentially expressed in the cortex and dentate gyrus based on the Allen Brain Atlas IHC dataset (48). Consistent with the known gene expression levels, we detected increased chromatin accessibility at Prox1 and Lhfpl2 loci in the dentate gyrus (*SI Appendix*, Fig. S6 *A* and *B*). Similarly, Pamr1 and Ddit4l loci exhibited higher accessibility in the cortex (Fig. 3*E* and *SI Appendix*, Fig. S6*C*). The peaks of accessibility overlapped with promoters and proximal or distal enhancer regions as defined by the modENCODE consortium (44), emphasizing the accuracy of the chromatin accessibility profiles we could obtain from this BALI-ATAC workflow. These data underscore the ability of BALI to profile chromatin accessibility across multiple spatial locations in intact tissues with a histology-driven approach.

### Spatial Multiomic Characterization of the Adult Mouse Hippocampus.

**Fig. 4.**
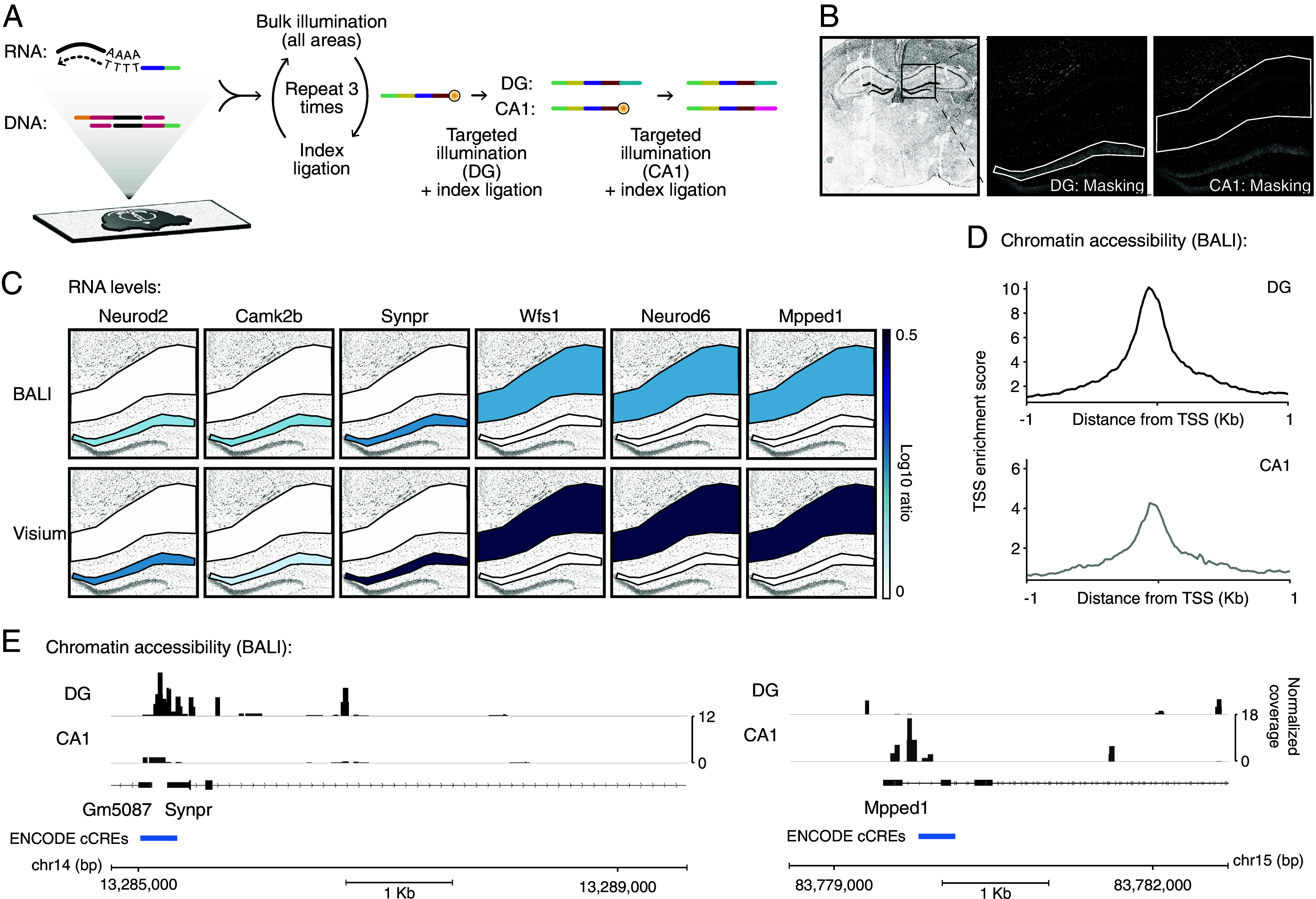
Multiomic profiling of gene expression and chromatin accessibility in the adult mouse hippocampus. (*A*) Schematic workflow. tissue sections from fresh frozen adult mouse brains were placed on glass slides, fixed, and permeabilized. In situ RT and template switching followed by Tn5 tagmentation with custom oligos were used to label the tissue mRNAs and accessible chromatin with the same BALI ligation root featuring a 5’P overhang (green). Three rounds of ligation and whole-slide illumination extended the root with three different indexes (yellow, purple, magenta) to encode the first, second, and third digits of a 4-digit spatial barcodes, with the last ligation producing a photocaged 5’P. The Dentate Gyrus (DG) and the CA1 hippocampal region (CA1) were sequentially illuminated and ligated with area specific indices (blue and orange). (*B*) Representative microscopy images for the tissue during uncaging (Draq5 stain). A coronal full section overview of the tissue section, with annotation of the field of view for uncaging (overlaid box). The close-up panels identify the illumination masks (white outline). (*C*) Gene expression of a curated list of markers. expression levels of three marker genes known to be upregulated in the DG (*Left*) and CA1 (*Right*) are shown in a scale of blue (log_10_ ratio) over the relevant illumination masks. *Top* row: BALI. *Bottom* row: results obtained from pseudobulking a previously published 10× Visium Dataset (49). (*D*) Enrichment at transcriptional start sites (TSS). The data are shown as cumulative plots (*Top*) for the DG (*Left*, black) and CA1 (*Right*, gray). The graph shown refers to 1 kb upstream and downstream of the TSS. Scales are adjusted for each area. (*E*) Representative coverage plots showing differential chromatin accessibility across two genomic loci, Synpr and Mpped1. Accessibility for the DG and CA1 are shown separately for each gene in black and gray, respectively. Gene annotations (RefSeq) and promoter annotations [blue, ENCODE cCRE (44)] are shown below.

To ask whether BALI could perform spatial multiomics, we next attempted to profile simultaneously the transcriptional and chromatin landscape across two distinct regions of the adult mouse hippocampus in thin tissue sections. Combining the transcriptome and chromatin profiling workflows explored above on the same tissue section, we first performed an in situ chromatin tagmentation step to install ligation roots on open chromatin, immediately followed by in situ reverse transcription and template switching to incorporate primers encoding the same ligation root in cDNA derived from polyA mRNAs (Fig. 4*A*).

Following tagmentation and reverse transcription, we performed iterative cycles of ligation and illumination to encode the appropriate spatial barcodes in either the dentate gyrus or the CA1 region of the hippocampus, with the same encoding scheme as described for our ATAC-seq experiment (Fig. 4 *A* and *B*). After sequencing, we were able to detect overexpression of Neurod2, Camk2b, and Synpr in the dentate gyrus and Wfs1, Neurod6, and Mpped1 in the CA1 (Fig. 4*C*), in line with publicly available data profiled with combined 10X Visium and scATACseq (49) (*SI Appendix*, Figs. S7 and S8). As observed in our single modality spatial ATAC-seq, chromatin accessibility at genic loci showed a peak immediately upstream of the TSS across all biological replicates (Fig. 4 *D* and *E*). Chromatin accessibility was differentially distributed in the two regions analyzed. Among the differentially regulated peaks, we confirmed differential chromatin accessibility for two loci for which we detected matched differential gene expression. First, we identified dentate gyrus-specific peaks in the promoter of Synpr, a gene encoding a synaptic protein known to be exclusively expressed in the mossy fiber of the dentate gyrus and CA2-CA3 of the hippocampus (50). Conversely, chromatin accessibility at the proximal enhancer of Mpped1 was restricted to the CA1 region (Fig. 4*F*). We mined our dataset further by identifying DNA motifs enriched in the ATAC peaks in each of the profiled regions (*SI Appendix*, Fig. S9). The dentate gyrus showed specific enrichment of motifs for several transcription factors involved in neurogenesis (i.e., NeuroD1, NeuroG2, E2FA, Sox15, Sox17), including critical regulators of granule cell development. We also detected some enrichment for stemness factors (Oct4), possibly reflecting the inclusion of a small portion of the hilus (a seat of adult neurogenesis) in our barcoded area. The CA1 region showed enrichment for factors involved in interneuron specification and regional specification (i.e., Dlx1, Dlx2, Lhx2, Gsx2). The two regions shared an enrichment in factors that have been shown to be generally present in the brain and hippocampus (50), including factors involved in genome organization (CTCF, Boris, YY1), constitutive transcription (Sp1, Sp5, Nfy), activity dependent plasticity (Jun, Fos), and neuronal development (Rfx1, Rfx2, Olig2, Atoh1, Atoh7). Overall, these data demonstrate the versatility of BALI for spatial profiling of tissues to collect multiomic information from a single sample.

### Automated Combinatorial Ligation on Solid Surface.

**Fig. 5.**
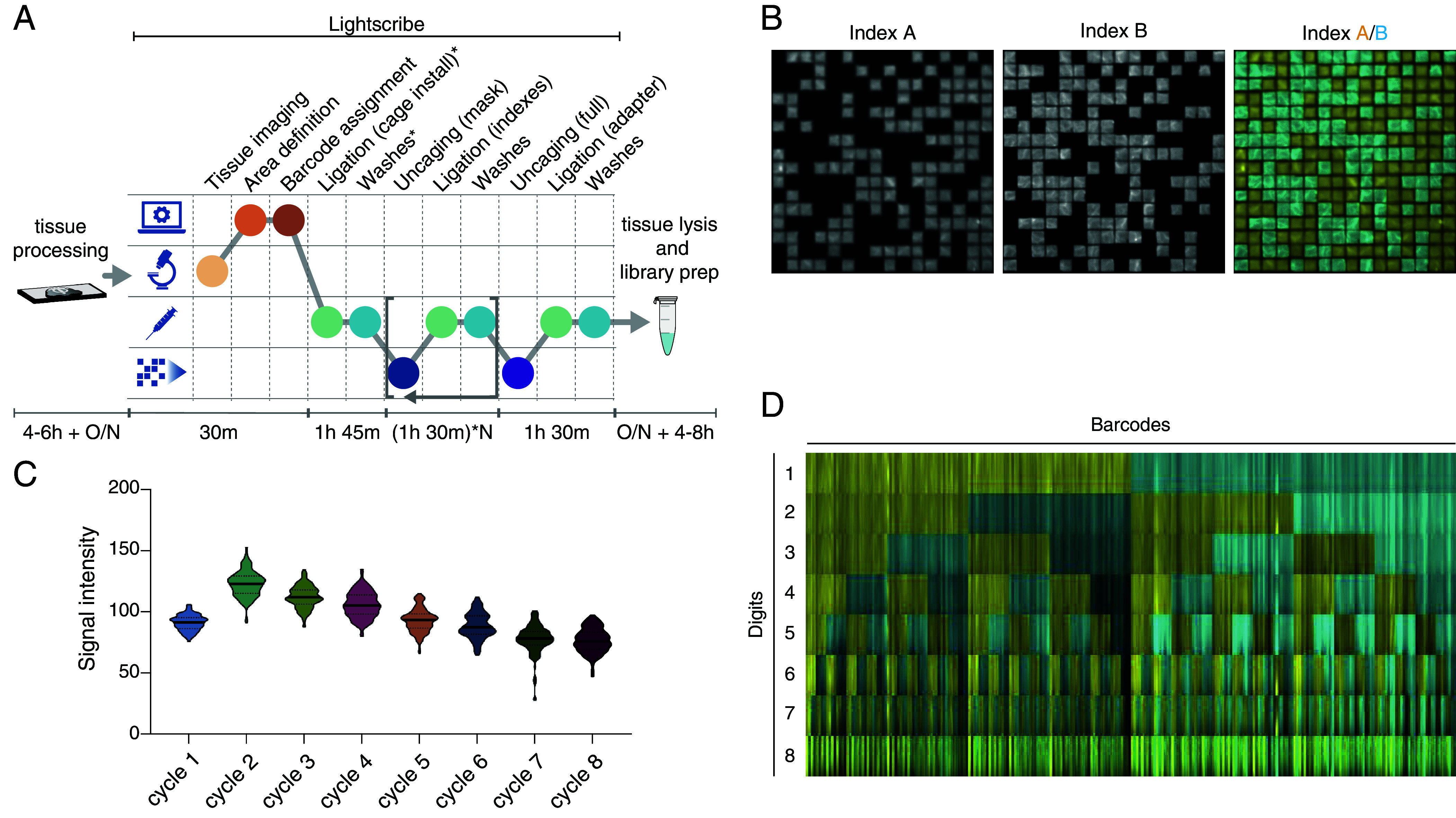
Automated combinatorial barcode writing on solid surface. (*A*) Schematic of the BALI process within the LightScribe instrument. After tissue processing (which installs a BALI root on the target biomolecule), a tissue section is imaged to identify regions of interest. Regions are then defined on the LightScribe software, and each is assigned a specific barcode. Optionally, a first ligation installs a bridge index bearing a photocage (if not already present on the root). Uncaging/until a full barcode is written in each region. A final ligation is performed to install a sequencing handle. The tissue is then taken out of the instrument and lysed to produce free DNA for amplification and library preparation. The color of each step represents their nature: yellow for imaging, orange/red for computational processes, teal/cyan for fluidics, and blue-violet for uncaging. (*B*) Example images for an automated experiment writing 256 barcodes on a functionalized slide. For this experiment, only two indices were used, writing an 8-digit barcode (complexity 2^8^ = 256). Each index bore a distinct fluorophore, cy5 or ATTO565. The images show the fluorescence signal for index A and index B after the first cycle of BALI, as well as their overlap showing sharp boundaries between regions. Each barcoded region is 100um in size. (*C*) signal uniformity across the whole profiled field of view for the 256 are barcoding experiment. Data are shown for index B only to avoid overcrowding of the image. Signal was quantified for each digit on a 20×20× cropped area. The violin plot indicates the average signal across all the areas positive for index B in each digit of the barcode. Signal is lower in cycle 1, peaks in cycle 2, and slowly drops over time as a consequence of gradually increasing errors in barcode writing. The plot also shows that uncaging and ligation efficiency are relatively uniform across the whole field of view, as indicated by the low variance of each column. (*D*) End result of 256 areas encoding. Composite image produced by overlapping fluorescence from index A and index B for each cycle (as in the rightmost image in (*B*) and stacking them to produce a representation of the whole barcode, with colors representing the index for each digit. Each of the 256 barcoded regions was cropped, reshaped by squeezing it to a thin rectangle, and displayed on the *x* axis of the composite image. The *y* axis represents time, as a proxy for each digit. This image provides a complete representation of the raw data for the entire barcoding experiment.

The recursive nature of combinatorial ligations is inherently amenable to automation, which further expands the scope of BALI from a handful of handpicked regions to large numbers of target areas. Here, we introduce a simple setup, which we named LightScribe, which enables fully automated barcode writing on solid surfaces, from functionalized slides to tissue sections. The LightScribe is a tripartite system composed of a low magnification microscope for optical access to the sample, a DMD projector to finely control illumination masks, and a robotic fluidics system to automate the molecular biology workflow. Upon imaging, dedicated software parses the image to design an illumination pattern based on features decided by user (e.g., grid vs. segmentation, number of areas, resolution). It then generates a barcode dictionary, assigns each spatial feature to a molecular barcode, and computes an integrated fluidics and illumination workflow to encode the spatial barcodes on the sample (Fig. 5*A*). The sample is clamped in a custom-built chamber with physical access to a needle controlled by a robotic arm, and optical access for imaging and illumination on either side of the sample. The light engine of the LightScribe can deliver 39 W/cm^2^ of light at 405 nm on a field of view of 3 × 5mm and with a resolution of 3 μm/pixel (as shown above). The fluidic system is designed to have zero dead volume and to require only ~300 μL of solution per reaction (assuming a flow cell with 20 mm diameter and 0.8 mm thickness), reducing the consumption of expensive reagents such as ligase. The device can perform a ligation cycle in approximately 90 min, which scales to ~33 h for a 20-cycle protocol capable of labeling 1,024 areas. The LightScribe is entirely built from affordable components, its most expensive element being the DMD projector (market price around $25,000 to $30,000). The total cost to build the system is approximately $45,000. Further specifications for the LightScribe are included in *SI Appendix*, Table S1.

To investigate the theoretical throughput of BALI, we used the LightScribe to spatially encode 8-digits barcodes on a solid support surface. As previously, we grew the molecular barcodes on a slide functionalized with a root oligo. Each digit of the barcode could have two different identities, “A” or “B” (Fig. 5*B*), which differed both in terms of DNA sequence and linked fluorophore (Atto565 and Cy5). Despite some increased heterogeneity with successive cycles, ligation efficiency was consistent across the entire field of view, with SD <10% of the total intensity values (Fig. 5*C*). We were able to detect all the 256 (2^8) different combinatorial barcodes through tracking of the fluorescent signal over cycles (Fig. 5*D* and Movie S3). This experiment demonstrates that automation allows scaling of BALI’s throughput to target hundreds to thousands of regions (depending on the number of indexed digits in the barcodes dictionary) enabling high-resolution profiling of tissue sections.

## Discussion

Here we have described BALI, a method for multiomic profiling of intact tissue sections via light-driven combinatorial ligation of DNA barcodes with user-defined resolution and throughput. BALI introduces a combinatorial encoding scheme that exponentially expands the throughput range from tens to millions of independent spatial locations–thereby theoretically enabling single cell analysis in situ.

Importantly, BALI can achieve subcellular-scale spatial resolution (~3 μm), which could be further increased by using higher-resolution optics in the DMD projector, albeit at the cost of a reduction in the illumination field of view and therefore speed. While other existing spatial omic methods exists in which spatially referenced barcodes are arrayed with high resolution (up to 2 μm for HDST or 10× Visium HD), BALI barcodes can be assigned computationally to areas defined by the user, which can allow for nonuniform shapes and sizes to capture unique and important features of histology and biological knowledge while minimizing redundancy. Combining light activation with combinatorial barcoding, BALI offers a solution to the conflict between resolution and throughput that has thus far dominated the field of spatial profiling. Together, these features enable BALI to offer both high-throughput profiling and histology-driven analysis of intact tissue samples.

The idea of combining photoactivation and sequencing to achieve spatially restricted profiling has been exploited by few other techniques, such as Light-Seq (29), photoselective sequencing (51), PHOTON (52), ZipSeq (53), photo-isolation chemistry (54), and geoMX (55). These are largely based on using light to either 1) control the binding of a poly-T probe to RNA or its 3’ extension, 2) control the hybridization of a barcode to a tag on the cell surface, followed by single-cell sequencing, 3) control the addition of a 5’ barcode to cDNA or tagmented DNA via uncaging of 5’ phosphate, as in BALI, 4) control crosslinking of a barcode to cDNA followed by cross-junction synthesis, or 5) control the release of tagged molecules from specific regions. A common limitation of these techniques is that they do not lend themselves easily to the production of long, multidigit combinatorial barcodes, either by design or because of technical features. For instance, in Light-seq, each element of the barcode is added by crosslinking, and the requirement for cross-junction synthesis during library preparation would likely result in decreased yield and increased errors as the number of junctions increase. As a result, the technologies mentioned above are largely capable of profiling only a few areas, typically lower than a hundred. On the other hand, BALI has been designed from the beginning with the idea of producing combinatorial barcodes capable of labeling thousands to millions of areas, and we demonstrated the ability to produce barcodes up to eight digits long.

The guiding principle behind BALI was to develop a profiling method that adapts to the biological question rather than forcing the question to fit the method. Our approach lets researchers tailor the resolution, profiling depth, and throughput within and across projects. This flexibility has two main implications: First, it enables biology driven profiling at lower resolutions, addressing the long-standing challenge of multicell signal deconvolution within individual spots. Second, by limiting profiling to relevant areas and only at the necessary resolution, it reduces experimental and sequencing costs. This is a key advantage for large cohort studies where current gold-standard methods remain prohibitively expensive for large and diverse patient sampling. Akin to the recent push toward large tissue sampling seen for commercial platforms, BALI readily scales up to large tissue sections–such as those of human samples–by simply tiling the area with multiple illumination masks, each adding only a minor fraction of the total run time. To fully realize BALI’s potential, further work will likely be required to optimize efficiency at each serial step, to refine the protocol to allow for cheaper reagents or lower their volumes, as well as to introduce strategies to profile archival FFPE samples (56–58). These advances would enable large longitudinal studies and to the vast repositories of biobanked specimens. Other approaches based on in-situ sequencing, for instance BARseq, have focused on maximizing throughput and reducing cost for large-sample RNA profiling, for instance profiling a whole mouse cortex (18). Compared to these, however, BALI has the advantage of being able to target individual areas specifically and to combine multiple omic readouts in the same experiment

A major limitation of state-of-the-art spatial multiomic methods is their reliance on bespoke equipment that is inaccessible to most labs. By contrast, BALI has two main requirements: 1) prompt tissue segmentation and barcode assignment prior to the barcode writing; and 2) precise patterning of light from a single source to parallelize barcode assignment to all the areas with the same digit *i* in position *n.* While tissue segmentation and barcode assignment are computationally intensive processes, the wider access to high-performance graphic cards as part of the AI revolution has turned this seemingly limiting step into a routine process that can be completed within minutes. And while laser scanning optical setups–such as a confocal microscope–are not well suited to simultaneous illumination of multiple tissue segments, digital micromirror devices (DMDs) offer the opportunity to precisely pattern the light emitted from a light source, thus illuminating all the regions of interest at once. In principle, any such device coupled to a microscope and to a fluidic system could be used to perform BALI at scale. In this paper, we present an example, based on relatively inexpensive and easy to obtain components, which combines a very large field of view with low reagent consumption, which minimize the time and cost required for full profiling experiments. While the LightScribe provides an integrated option capable of automating the whole protocol, it should be relatively easy to adapt only specific components of our setup (i.e., the fluidic subsystem) to integrate them with existing illumination setups, or to tweak them for specific applications. Therefore, we believe that BALI has the potential to provide a significant improvement in accessibility to spatial omic technologies.

Nucleic acids are the natural targets of any method based on a sequencing readout. However, there is the potential to profile a broader portfolio of biomolecules by incorporating oligonucleotide-functionalized libraries of antibodies (27, 59). Antibodies can detect a broad spectrum of biomolecules (e.g., proteins, posttranslational modifications, metabolites, lipids), yet they often suffer from poor specificity and limited resilience to oligonucleotide functionalization. We envision that the expanding catalog of single chain antibodies (60) and the development of orthogonal chemistries to specifically decorate target molecules with oligonucleotides [e.g., aptamers (61)] will significantly extend the scope of spatial multiomic profiling abilities in future work. We believe that complementary innovations on multiple fronts are now coalescing to expand the applicability of spatial multi-omic technologies to new sample types and readouts–all of which should be compatible with BALI.

Considered as a whole, BALI conceptually overcomes many limitations in the spatial multiomics field, providing a high-resolution, adaptable, and accessible platform for optical, histology-informed barcoding that is poised to capitalize on emerging technical advances to further increase the scope, resolution, and applicability of spatial multiomic profiling.

## Methods

Extended methods are available in the *SI Appendix*. Sequences for all oligonucleotides are provided in *SI Appendix*, Table S2.

### Animal Procedures.

All animal procedures were performed in accordance with the Animal (Scientific Procedures) Act 1986 under UK Home Office project license PAD85403A.

### BALI Ligation Cycles.

DNA indices were produced by annealing complementary oligonucleotides as per extended methods and *SI Appendix*, Table S2. Ligations were performed for 30 to 60 min using T4 DNA ligase (1:20) in a buffer containing 2.7 uM indexes, 1× T4 ligase buffer. Slides were washed in 2× SSC 10’, 2× SSC+10% ethylene carbonate (5’) and 2× SSC (5’), followed by a quick wash in 1× T4 ligation buffer if a new ligation was to be performed

### Barcode Writing on Slides.

Experiments were performed on NH2-functionalized slides (Sigma) or slides coated in streptavidin-doped acrylamide (as per extended methods). Uncaging was performed on a Leica SP5 confocal microscope (15 min) or on the lightscribe instrument (2 min). After BALI ligations slides were imaged on an epifluorescence microscope

### Barcode Writing on Beads.

Ligations were performed on Sepharose NHS beads functionalized with NH2-modified anchor oligos as per extended methods and *SI Appendix*, Table S2. A fluorescent root oligo with a free 5’P was hybridized to the anchor. Ligated products were denatured from the beads by boiling the beads at 95 °C for 5 min in 1× RNA loading dye and run on a 15% TBE-Urea gel.

### Barcode Writing on Tissue.

Ligations were performed on 10 μm cryosections from the adult mouse liver. Sections were fixed and permeabilized as per extended methods. A poly-T oligonucleotide with a 5’ anchor sequence was hybridized to cellular mRNAs in conditions mimicking a RT reaction. A fluorescent BALI root with a 5’P was then hybridized to the anchor. Ligated products were purified and run on a gel as above.

### BALI-RNA.

Coronal cryosections from E16.5 mouse brains were fixed and permeabilized as per extended methods. A poly-T BALI root oligonucleotide with a 5’ photocage was hybridized to the tissue and reverse transcription/template switching performed in situ overnight. Sections were then washed, stained with Draq5, and imaged. Target regions were identified and uncaged using the 405 nm laser of a Leica SP5 microscope for 5’. After BALI barcoding, tissue was lysed overnight at 60 °C in RIPA buffer+proteinase K. DNA was purified from the lysate using and amplified with indexed Truseq oligos to produce sequencing libraries.

### BALI-ATAC.

Coronal cryosections from the adult mouse brain were lightly fixed and permeabilized as per extended methods. A custom set of transposition adapters with a 5’ BALI root was loaded on tn5 transposase and used to tagment accessible DNA in situ. A first ligation was performed to install a photocaged bridge on the root, followed by three BALI ligations in bulk (uncaging performed on a transilluminator), area-specific uncaging, and a final ligation. After BALI barcoding, tissue was lysed and DNA was purified and used to produce ATAC sequencing libraries.

### BALI-Multiome.

The protocol was performed as described above for BALI-RNA and BALI-ATAC. Tissue was fixed and tagmented as per the ATAC protocol, using a biotinylated set of adapters. Reverse transcription was then carried out as per the BALI-RNA protocol. This produced accessible DNA fragments and cDNA bearing the same BALI root. After BALI barcoding, tissue was lysed, and the purified DNA added to streptavidin beads to separate tn5 fragments (pull-down) from cDNA (supernatant). The two fractions were then amplified separately to produce ATAC and RNA-Seq libraries.

### LightScribe Instrument.

The LightScribe instrument is a custom-built microscope featuring three subsystems: Uncaging (composed by a high-power 405 nm commercial LED DLP projector), Imaging (a basic epifluorescence microscope setup with multichannel imaging capability and a 4× objective), and Fluidics (composed by a robotic arm connected to a syringe driven by a peristaltic pump). This setup can automate multiple cycles of uncaging and ligations, simplifying the execution of complex multistep BALI experiments. Full details are in the extended methods.

## Supplementary Material

Appendix 01 (PDF)

Dataset S01 (DOCX)

Movie S1. Video outlining the BALI combinatorial ligation process, in which the step-wise addition of DNA indices to a growing chain (triggered by light-activation) results in the production of a complex multi-unit spatial barcode.

Movie S2. Combinatorial barcode writing for 16 areas (2 indices / 4 cycles - main text Fig. 1c). The video was generated by taking a multichannel (FITC / cy5) confocal microscope image after the ligation of the final index for each cycle. Brightness/contrast for each image were adjusted to make the signal intensity consistent. Images are in pseudo-colors in a color-blind friendly palette.

Movie S3. Combinatorial barcode writing for 256 areas (2 indices / 8 cycles – main text Fig. 5). The video was generated by taking a multichannel (ATTO 565 / cy5) confocal microscope image after the ligation of the final index for each cycle. Brightness/contrast for each image were adjusted to make the signal intensity consistent. Images are in pseudo-colors in a color-blind friendly palette.

## Data Availability

All the sequencing data are available through GEO (accession no. GSE260699) (62). Raw images and non sequencing data are available from the authors upon request. The scripts used for analysis and data processing are available from the authors upon request. Similarly, the LightScribe software is available from the authors upon request.
