# Supplementary material for: Spatially tunable multiomic sequencing using light-driven combinatorial barcoding of molecules in tissues
Source: Proc Natl Acad Sci U S A. 2026 May 18;123(21):e2527896123. doi: 10.1073/pnas.2527896123 (PMC13214022; doi:10.1073/pnas.2527896123)
Supplement: Supplementary file 1 — Appendix 01 (PDF) [file pnas.2527896123.sapp.pdf]

PNAS

# Supporting Information for:
## Spatially tuneable multi-omics sequencing using light-driven combinatorial barcoding of molecules in tissues

*Giorgia Battistoni[1,a], Sito Torres-Garcia[1,a], Chee Ying Sia[1,+], Silvia Corriero[1], Carla Boquetale[1], Elena Williams[1,+], Anna V Cregeen[1], Karolina Wasilewska[1], Martina Alini[1], Nicole Hemmer[1], IMAXT Cancer Grand Challenge Consortium, Shankar Balasubramanian[1,2], Benjamin Czech Nicholson[1], Gregory J. Hannon[1,*], Dario Bressan[1,*]*

[1] CRUK Cambridge Institute, University of Cambridge, Cambridge (UK)
[2] Department of Chemistry, University of Cambridge, Cambridge (UK)
[a] These authors contributed equally to this work

* Corresponding authors
+ current address: MRC Laboratory of Molecular Biology, Cambridge (UK)

Corresponding authors: Gregory J. Hannon, Dario Bressan
Email: Greg.Hannon@cruk.cam.ac.uk , Dario.Bressan@cruk.cam.ac.uk

**This PDF file includes:**

Extended Materials and Methods
Figures S1 to S9
Table S1 to S2
Legends for Movies S1 to S3
Consortium authors list

**Other supporting materials for this manuscript include the following:**

Movies S1 to S3

## Extended Materials and Methods

### Overhang screen

Eight oligonucleotides (BL354 to BL357, and BL354B to BL357B) were synthesized with normal desalting purification. The oligos were annealed to produce two double-stranded DNA molecules (A and B) containing a constant region corresponding to the Truseq P5 and P7 sequences plus a multiplexing sequencing index, and a random 6-mer or 7-mer sequence protruding at the 5' end (ligation overhang). Oligos were annealed at 30 μM concentration in 2X SSC by running the following program in a thermocycler: 1) 95°C for 5 minutes, 2) ramp down to 65 at 0.1°C /second, 3) hold at 65°C for 5 minutes, 4) ramp down to 12°C at 0.1°C/second, 5) hold at 12°C. The A and B dsDNA molecules for each overhang length (6-mer or 7-mer) were diluted to 3.334 μM in 2X SSC and used to assemble a ligation reaction as follows: 2 μL 10x T4 ligase buffer, 1 μL of each annealed dsDNA (final 166.7 nM), 1 μL T4 DNA ligase (NEB M0202M), 15 μL nuclease-free water. Ligation was performed for 3 minutes at 22°C , following by heat-inactivation at 65°C for 10 minutes. The ligated molecules were purified by running the whole reaction volume on a 2% TBE agarose gel. The gel was stained in 1:10000 SYBR-gold (Thermo Scientific) and the bands corresponding to the ligation products were purified using the Qiagen Qiaquick gel extraction kit using the supplier's standard protocol. Library concentration was estimated by Tapestation (DNA5000 screentape) and qPCR (KAPA Illumina library quantification kit).

The fastq files containing raw sequencing reads were first filtered using the cutadapt package (v4.1) using the following options: -a AGATCGGAA -m [length of overhang] -M [length of overhang +1]. This extracted the sequence corresponding to each ligated 6-mer or 7-mer overhang in the library. The resulting files were processed through a custom python script counting the number of occurrences of each overhang, producing a final .csv file with overhang counts.

### Overhang screen validation

DNA indexes with different overhang sequences were validated by performing in-situ ligation assays on 4T1 mouse cells grown on 25mm round coverslips coated in poly-L-lysine overnight (Sigma P4707, 0.01% w/v). Cells were grown for 24-48hrs to ~90% confluence.

Indices for each overhang to be tested, as well as the BL092_50nt root oligo, were ordered as oligonucleotides with a 5' phosphate on the overhang subjected to ligation. For each overhang to be tested, two indices were annealed. BL407 to BL426 (6-mers) or BL452 to BL471 (7-mers) were annealed to BL427 to extend the root with the overhang 'XX' to be tested ('Index_A_XX'), whereas BL428 to BL447 (6-mers) or BL472 to BL487 were annealed to BL448 to generate a reverse complement overhang to the one tested ('Index_B_XX'). To increase phosphorylation yield, oligos with 5' phosphate were further phosphorylated by incubating them (at a concentration of 50 μM for indices and 25 μM for BL092-50nt) with 10U T4 polynucleotide kinase (NEB) in 1X T4 DNA ligase buffer (NEB). The reaction was carried on for 30 minutes at 37°C and stopped by heating to 60°C for 20 minutes, followed by purification through Illustra Microspin G-25 columns (Cytiva) as per supplier's protocol. DNA indices were annealed to a final concentration of 20 μM in 2X SSC using the following protocol in a thermal cycler: 1) 95°C for 5 minutes, 2) ramp down to 65 at 0.1°C /second, 3) hold at 65°C for 5 minutes, 4) ramp down to 12°C at 0.1°C /second, 5) hold at 12°C.

Cells were fixed in 4% PFA/PBS for 15 minutes at RT, washed in PBS, and permeabilized in 1% Triton X-100 in PBS for 15 minutes at RT. After permeabilization, cells were further cleared and permeabilized by incubating them in 0.3% SDS in PBS for 30 minutes at RT, followed by three washes in PBS for 10 minutes each. Hybridization of the root was performed by inverting the coverslips on a drop (25 μl) of 0.1 μM polyT-rootV2 oligo diluted in hybridization buffer (2x SSC, 1 mg/mL yeast RNA, 10% dextran sulfate, 1:1000 Murine RNase inhibitor NEB M0314) on a parafilm sheet placed on a glass plate. Hybridization was performed for 24hrs at 37°C in a humidified chamber. The following day, coverslips were transferred to a 6-well plate and washed three times with 1 mL of 2X SSC for 5 minutes each and once in secondary hybridization buffer (2X SSC, 10% Ethylene Carbonate) for 5 minutes, all at RT. Samples were then hybridized with 2 ml of 1.25 nM

BL092-50nt oligo in secondary hybridization buffer for 20 minutes at RT, followed by three more washes in 2X SSC for 5 minutes each.

A ligation reaction was then assembled by combining 2 μl 10x T4 DNA ligase buffer (NEB), 1 μl annealed 20 μM Index_A_XX stock, 16ul nuclease-free water and 1 μl T4 DNA ligase (NEB). The entire volume of the reaction was pipetted on a parafilm sheet placed on a glass plate and the coverslips were inverted over it. Ligation was carried out at 25°C for 45 minutes in a humid chamber. After the ligation, coverslips were washed 3 times in 2X SSC for 5 minutes each to remove any unligated indices. A second ligation was performed as above using the cognate Index_B_XX.

Ligated root molecules were eluted by pre-heating plastic surface (the lid of a 6-well multiwell plate) at 95°C on a thermoblock. 50 μl of pre-heated 2X RNA loading buffer (95% deionized formamide, 5% 10x TBE, 5mg bromophenol blue) were pipetted on the surface and the coverslips were inverted on them. Samples were incubated for 10 minutes at 95°C, after which the coverslips were carefully lifted, and the liquid collected under them collected with a 200μm pipette (approx. 30 μl were recovered per sample) and transferred to a 1.5ml Eppendorf tube. Samples were further incubated at 95°C for 10 minutes in a thermoblock and loaded on a 6% TBE-Urea acrylamide denaturing gel (Thermo Scientific). Gels were stained with SYBR-gold (1:10000 in 1x TBE, Thermo Scientific) and imaged using an Amersham Typhoon instrument in the cy2 and Cy5 channels. Ligation efficiency was evaluated by densitometry on the Cy5 gel images.

**Acrylamide slide preparation and coating with BALI root oligos**

Slides were functionalized by adhering to them a thin polyacrylamide gel exposing streptavidin molecules, and bound to a carpet of biotin-modified root oligonucleotides capable of being ligated to BALI indices. Glass slides were first coated with 0.05% v/v BIND-silane (3-(Trimethoxysilyl)propyl methacrylate - - Sigma M6514) diluted in 80% EtOH / 2% Acetic acid for 1h at room temperature, and washed in Ethanol before being air-dried. Two strips of scotch tape were used at the beginning and end of the slide to create spacers allowing the formation of a ~50 um thick gel. A solution of 4% 19:1 acrylamide-bisacrylamide (Bio-Rad 1610144), in 50 mM Tris-HCl pH 8 / 300 mM NaCl was degassed under vacuum and polymerization was initiated by addition of 0.1% ammonium persulfate and 0.1% N,N,N′,N′ -Tetramethylethylenediamine (TEMED). 160ul of the polymerizing acrylamide solution were quickly mixed with 40ul of 2mg/ml streptavidin-acrylamide (Thermo Scientific S21379) diluted in 1X PBS, and 100ul each were pipetted in the center of two glass plate pre-treated with gel-slick solution (Lonza 50640). The bind-silane activated slides were inverted on the drop of acrylamide solution and left to polymerize for 1h before being lifted with a razor blade. A thin acrylamide gel was left on the slide surface. Acrylamide slides were washed twice in 1X PBS, then 20ul of a 10 uM solution of BL-940 oligonucleotide (biotin-modified BALI root) were pipetted (in a dark room) in the center of the gel, covered with a 12mm round coverslip, and incubated for 30 minutes in the dark in a humid chamber. Slides were then washed twice for 5 minutes each in 2X SSC and stored in SSC until required for the barcode writing experiment.

**Barcode writing on slide**

Amine coated glass slides (Silane-Prep, Sigma, S4651) were washed in 70% Ethanol and air dried. All furthers steps were performed in light protected conditions. A functionalization mix (50 μL) was prepared fresh with the 3'aminated 5' caged oligo root carrying a terminal Cy3 dye (2 μM BL003 for the single-cycle experiment and 2 μM BL728 for the 4-cycle experiment) and a bifunctional crosslinker BS(5)PEG (Thermo Scientific) (5 μM in dimethylformamide), and placed at the center of the slide as a drop. The functionalization was run 16 hrs at 37C in a humidified chamber. The functionalized slides were washed twice in 2X SSC (Thermo Scientific), and then mounted with 200 μL of 2x SCC and a thin glass coverslip (22x22mm).

For the single-cycle experiment in supplementary figure 2, uncaging was performed on a SP5 Leica confocal system fitted with a 30mW 405nm solid state laser, illuminating for 2 minutes ("1" shape) and 5 minutes ("2" shape) with the 405nm laser at maximum power through a 10X objective. After uncaging the slide was imaged in the cy3 channel using the 514nm laser line to detect the removal of the fluorophore in the uncaged areas. The slide was then washed 3 times for 5 minutes each in 2X SSC. Oligos *lig_test_onbridge1_fw** and *lig_test_onbridge1_rev* were annealed by mixing them at 45 μM final concentration in 2X SSC and running the following program in a thermocycler: 1) 95°C for 5 minutes, 2) ramp down to 65 at 0.1C/second, 3) hold at 65C for 5 minutes, 4) ramp down to 12C at 0.1C/second, 5) hold at 12°C. A ligation mix was then prepared with the annealed index oligos (2.7 μM), T4 ligase (100 U/μL, NEB) in 1x DTT free ligation buffer in a final volume of 50 μL. Any excess liquid from previous washes was removed from the slides, and 50uL of ligation mix were added to the marked uncaged area, covered with a coverslip and incubated for 60 minutes at 25°C in a humid chamber. After ligation, the slide was washed in 2X SSC for five minutes three times, in 0.2X SSC for five minutes once, and then in 0.2X SSC overnight. The following day the slide was imaged in the cy3 and cy5 channels using the 514nm and 633nm laser lines to detect the cy5-positive ligation products.

For the 4-cycle experiment, uncaging was performed as above illuminating for 15 minutes with the 405nm laser at maximum power. The sixteen barcode areas were first defined as separate square ROIs, then selected appropriately for each uncaging cycle. After uncaging, the slides were imaged as above (cy3 and Cy5 channels) and then washed for 5 minutes in 2x SSC, 5 minutes in 0.2x SSC, and 5 minutes in 1x DTT free ligation buffer (50 mM TrisHCl pH7.4, 10 mM $MgCl_2$, 1 mM ATP). The index oligos for each ligation were annealed as above (BL736/BL641, BL737/BL644, BL646/BL642, BL647/BL643). The ligation mix was prepared with the annealed index oligos (2.7 μM), T4 ligase (100 U/μL, NEB) in 1x DTT free ligation buffer in a final volume of 50 μL. Any excess liquid from previous washes was removed from the slides, and 50 μL of ligation mix were added to the marked uncaged area, covered with a coverslip, and incubated for 60 minutes at 25°C in a humid chamber. After ligation, the slides were washed 10 minutes in 2x SSC, 5 minutes in 2x SSC + 10% Ethylene Carbonate, 5 minutes in 2x SSC, then mounted with 200 μL of 2x SSC for the additional round of uncaging and imaged in the cy3, cy5 and FITC channels.

For the resolution measurement experiment in figure 1, acrylamide-modified slides coated with the root oligos were prepared as described in the section above. The slide was then clamped in the flow-cell of the automated LightScribe instrument (described below in the methods) and, after focusing, illuminated with an uncaging profile featuring a regular grid alternating lines of 1px and 2px thickness, as well as 1px dots. Illumination was performed for 2 minutes with a current setting of 10,000 mA, corresponding to a light flux of approx 0.39 W/mm$^2$ and to a total delivered energy of ~47 J/mm$^2$. After uncaging, the slide was removed from the lighscribe device and washed once for 10 minutes in 2X SSC, once for 5 minutes in 2X SSC / 10% ethylene carbonate, and once for 5 minutes in 2X SSC. After a final wash in 1X T4 ligase buffer for 5 minutes, ligation was performed for 1h using oligo pair BL646/BL642 using the same protocol described above, with the difference that regular 1X T4 ligase buffer was used (including DTT). After ligation, the slide was washed further once in 2X SSC for 10 minutes, once in 2X SSC / 10% ethylene carbonate for 5 minutes, and once in 2X SSC for 5 minutes, before imaging.

All experiments were imaged on a Leica Dmi8 microscope equipped with a solid-state white light source (Lumencor SOLA) and filter sets for DAPI, FITC, TRITC and cy5.

**Resolution and contrast measurement**

Uncaging/ligation resolution measurement was conducted on a slide uncaged in a grid/dot pattern as described in the section above. All analysis was conducted using the *fiji* software. First, an intensity profile was obtained across either five of the 1px lines or five of the 1x dots in the image. A gaussian function was then fitted to the profile, and the full-width at half maximum of the peak was calculated after correcting for background signal.

Contrast was calculated by measuring average signal intensity for five uncaged areas and for five non-uncaged areas within the projector field of view, and calculating the ratio between the two after subtracting signal background obtained from five "dark" regions outside the projector field of view.

### Multiple ligations on beads

Sepharose NHS beads (50 μL of resin bed) were washed twice in 20 volumes of 1 mM HCl, and twice in 20 volumes of 2x Coupling Buffer (100 mM Sodium Borate pH 8.5). After the last wash, the resin bed was resuspended in 2 volumes of 50 μM of 5' amino modified oligo (PolyT_root_probe_v2). The beads were functionalized for 4 hrs at room temperature with constant rotation. The reaction was blocked by adding 20 μL of 1M Tris-HCl pH 8 and rotating the beads for additional 30 minutes at room temperature. The beads were washed four times for 5 minutes in 100 mM Tris-HCl pH 8 and either used immediately or stored in 1x Storage buffer (100 mM Tris-HCl pH 8, 2.5 mM EDTA) for up to 1 month at 4°C. Prior to the ligations, all oligos purchased with a 5' phosphate were additionally enzymatically phosphorylated with 0.2 U/μL T4 PNK, 1 mM ATP, 0.5 μM 5'P oligo in 1x PNK reaction buffer for 30 minutes at 37°C, and then heat-inactivated for 20 minutes for 60°C. Samples corresponding to a different number of serial ligation cycles were processed in parallel, removing one sample at each cycle for analysis. For each sample, 12.5 μL of functionalized bead resin were washed twice in 1x Hybridization buffer (2x SSC, 10% Ethylene Carbonate in H20) and hybridized with 100 μL of a 3' Cy5 labelled 5'P oligo root (BL92) (1 μM in 1x Hybridization buffer) for 30 minutes at room temperature with constant rotation. To remove any free fluorescent oligo, the beads were washed 3 times for 10 minutes in 1x Hybridization buffer, and 2 times for 5 minutes in 2x SSC with constant rotation. The index oligos for the ligations were annealed at 45 μM final in 2x SCC in the following pairs: BL46-PNK/BL45 (cycles 1, 3, 5, 7) and BL47-PNK/BL48 (cycles 2, 4, 6). The annealing thermocycler conditions were as follows: 95°C for 5 minutes, ramp down to 65°C at 0.1°C /sec, 65°C for 5 minutes, ramp down to 4°C at 0.1°C /sec, hold at 4°C. For each cycle of ligation, a ligation mix (20 μL) was prepared with annealed index oligos (2.7 μM) alternating BL46/45 and BL48/47, T4 ligase (100U/μL, NEB) in 1x T4 ligation buffer in a final volume of 20 μL and added directly to the beads. The ligation reactions were incubated for 30 minutes at room temperature with constant rotation, and then washed 3 times for 5 minutes in 2x SSC. After the serial ligations, the ligated products were denatured from the beads by boiling the beads at 95°C for 5 minutes in 1x RNA loading dye (47.5% formamide, 0.01% SDS, 0.01% bromophenol blue, 0.5 mM EDTA). The beads were centrifuged and the supernatant run on a 15% TBE-Urea gel. Images of the gels were acquired with an Amersham Typhoon.

### Animal housing

All animal procedures were performed in accordance with the Animal (Scientific Procedures) Act 1986 under UK Home Office project license PAD85403A. Animals were housed under standard husbandry conditions per UK Home Office and Institute regulations. Individually ventilated cages housed four to five animals under a 12-h light, 12-h dark cycle at the CRUK Cambridge Institute animal facility. Relative humidity was kept between 45 and 65% with a temperature range of 20-24°C. Animals had free access to water and pelleted food. Embryos samples were taken at embryonic day 16.5 from ethically euthanized pregnant dams following timed mating. Adult samples were collected from ethically euthanized 8-12 month-old animals.

### Tissue collection and Histology

For embryo collection, pregnant dams were euthanized by CO2 overdose with exposure to gas in rising concentration followed by cervical dislocation. Embryos were euthanized by cooling on ice followed by decapitation. Heads were frozen in OCT resin by immersion in a dry ice/isopentane bath followed by storage at -80°C. For adult brain collection, mice were euthanized by cervical dislocation followed by confirmation of death by permanent cessation of circulation. Animals were

then decapitated, and the brain dissected and frozen in OCT resin and stored as above. Cryosections were cut using a Leica CM3050S cryostat under RNase free conditions. The instrument was wiped with RNase-ZAP prior to use and between different blocks. A different blade was used for each block. Sample blocks were equilibrated to the chamber temperature for 15 minutes prior to sectioning, and then mounted on the object holder. Sections were cut at a thickness of 10 μM and collected on RNase-free SuperFrost Plus microscope slides (one section per slide). Slides were left at room temperature for 20-30 seconds to help tissue adhesion and then stored on dry ice (short term) or at -80°C (long term). Sections to be processed in the Laser-Capture microdissection workflow were collected on PEN membrane glass slides (Leica) and processed/stored similarly.

**Multiple ligations on tissue**

All steps were performed under RNase-free conditions. Cryosections of adult liver were retrieved from cold storage and equilibrated for 5 minutes at room temperature and then heated for 5 minutes at 37C on a metal heat block. Pap-pen was used to draw an hydrophobic boundary around each section, and let dry for 5 minutes at room temperature. Sections were incubated with 500 μL PBS to dissolve and remove the OCT embedding matrix and fixed with 500 μL of 0.2% PFA in PBS for exactly 5 minutes at room temperature. Fixation was quenched by removing the PFA and promptly adding 1.25 M Glycine in PBS, one quick wash and one wash for 5 minutes at room temperature. After washing twice in 1x wash buffer (20 mM HEPES pH 7.5, 150 mM NaCl, 0.5 mM spermidine, 1x proteinase inhibitor cocktail) for 5 minutes each, the sections were permeabilized in Permeabilization buffer (0.01% IGEPAL, 0.01% Digitonin in 1x Wash buffer) for 5 minutes at room temp, and then washed in 1x Wash buffer twice for 5 minutes. The permeabilized section were heated for 5 minutes in pre-warmed 1x PBS at 65°C in the hybridization oven, and then snap-cooled in ice-cold PBS on ice for 10 minutes. For each slide, a mock RT mix was prepared as follows to hybridize a root for sequential ligations. A first mix (14 μL) containing 2.5 μL of dNTPs (stock 10 mM each), 1.5uL of poly-T root (100 μM stock) in water was denatured for 5 minutes in 65°C and the snap cooled on ice for >2 minutes. A second mix (36 μL) was prepared with 10 μL of 5x SuperScript-IV buffer, 1 μL of RNAsin, 2.5 μL of 100 mM DTT in nuclease free water and added to the first mix. Any excess PBS was removed from the slides, and 50 μL of mock RT mix was added directly on top of the sections, which were then covered with a 22x22 mm coverslip and incubated overnight (~16hrs) at 42°C in a sealed humid chamber. Prior to the ligations, all oligos purchased with a 5' phosphate (BL611, BL604 and BL603) were additionally enzymatically phosphorylated with T4 PNK (1.6 U/μL), ATP (1 mM), 5'P oligo (50 μM) in 1x T4 reaction buffer for 60 minutes at 37°C and then heat-inactivated for 20 minutes for 60°C. The oligos were purified through a G25 resin column following the manufacturer standard protocol. The index oligos for the ligations were annealed at 30 μM final in 2x SCC in the following pairs: BL604-PNK/BL585 (cycles 1 and 3) and BL603-PNK/BL583 (cycle 2). The annealing thermocycler conditions were as follows: 95°C for 5 minutes, ramp down to 65°C at 0.1°C /sec, 65°C for 5 minutes, ramp down to 4°C at 0.1°C /sec, hold at 4°C. The slides were retrieved from the mock RT reaction, and washed twice in PBS, once in 2x SSC and once in 2x SSC + 10% EC for 5 minutes each at room temperature. The fluorescent ligation root was hybridized to the poly-T root by incubating the sections with 500 μL of 12.5nM BL611-PNK in 2x SSC + 10%EC for 30 minutes at room temperature. The sections were washed 3 times in 2x SSC and used for serial ligations. For each number of cycles, we processed two slides in parallel as replicates. For each cycle of ligation, a ligation mix (25 μL) was prepared with annealed index oligos (3 μM, alternating BL604/585 and BL603/583 as appropriate), T4 ligase (100 U/μL, NEB) in 1x T4 ligation buffer, added directly to the sections and covered with a 22x22mm glass coverslip. The ligation reactions were incubated for 30 minutes at 25°C in a humid chamber, and then washed 3 times for 5 minutes in 2x SSC. After the serial ligations, the ligated products were denatured from the sections by boiling the slides at 95°C for 15 minutes in 100uL of 1x RNA loading dye (47.5% formamide, 0.01% SDS, 0.01% bromophenol blue, 0.5 mM EDTA) on a flat heat block under humid conditions. The RNA loading dye was retrieved (~80uL) in a PCR tube,

additionally denatured for 10 minutes at 95°C and run on a 8% TBE-Urea gel. Images of the gels were acquired with an Amersham Typhoon.

In the survey of tissue fixation conditions, the fixation step was changed to 0.5% PFA in PBS for exactly 5 minutes, 4% PFA in PBS for exactly 5 minutes, or 4% PFA in PBS for exactly 15 minutes. An additional condition included a first fixation with 0.5% PFA in PBS for exactly 5 minutes and subsequently a post-fixation in chilled (-20C) 70% ethanol for 7 minutes. In the survey of ligation additives, the following were added to the ligation mix: 4% final Bovine serum albumin (molecular biology grade), 0.01% final Triton X-100, or 100 μg/mL yeast tRNAs. For these experiments, only one cycle of ligations was performed.

**Laser Capture microdissection on E16.5 mouse brain**

Tissue sections from E16.5 mouse embryos (as described in the "Animal housing" section) were cut as described in the "Tissue collection" section. Sections were stained with Haematoxylin/Eosin using the following fast procedure: 1) 40 seconds in 75% ethanol, 2) 30 seconds in mQ water, 3) 30 seconds in Harris' modified Haematoxylin solution (Sigma HHS16), 4) 3x 30 seconds in mQ water, 5) 30 seconds in a 1:1000 dilution of 28% Ammonium Hydroxide (Sigma 338818) in mQ water, 6) 10 seconds in 5% Eosin Y solution (Sigma 318906), 7) 30 seconds each in 70%, 95% and 100% ethanol, 7) 2x 20 seconds in Xylene. The stained sections were imaged and cut on a Leica LMD6000 LCM microscope. Areas corresponding to the sub-ventricular zone and to the cortex (matching the illumination areas for the BALI experiment) were used for cutting. Tissue fragments were collected in the caps of 0.5ml RNase-free tubes in 50 μl of lysis solution from the RNAqueous microRNA extraction kit (Thermo Scientific). RNA extraction was performed according to the RNAqeous micro kit protocol for LCM extraction. In short: 1) 50 μl of lysis buffer were added to the 50 μl including the sample, and the tube was incubated at 42°C for 30 minutes, 2) the collection column was pre-wetted with 30 μl lysis solution, 3) 3 μl LCM additive were added to each sample, 4) 129 μl of 100 μl Ethanol were added to each sample, 5) the samples were bound to the collection column, and the column was washed with 180 μl wash solution 1 and twice with 180 μl of wash solution 2/3, 6) RNA was eluted with a 5 minute incubation step with 10 μl of pre-heated (95°C ) elution solution. Purified RNA was treated with DNase using the optional DNase treatment step in the RNAqueous kit: 1) 1 μL of DNaseI buffer and 1 μL of DNase enzyme were added to each sample, 2) samples were incubated for 20 minutes at 37°C, 3) 2 μL of DNase inactivation reagent were added to each sample, and the inactivated DNaseI was removed by centrifugation. RNA abundance and quality were evaluated by Agilent Tapestation (DNA5000 High-Sensitivity screentape). RNA sequencing libraries were prepared using the NEBNext Single Cell/Low Input RNA Library Prep Kit for Illumina (NEB) according to the recommended protocol using 1 ng RNA as input material. Library quality and abundance was measured by Tapestation (DNA5000 screentape) and qPCR (KAPA Illumina library quantification kit). Libraries were sequenced on a NextSeq500 instrument using paired end 75nt reads.

**In situ RNA-seq**

*Tissue preparation and RT:* All steps were performed under RNase-free conditions. Cryosections of embryonic E16.5 brain (coronal plane) were retrieved from cold storage and equilibrated for 5 minutes at room temperature and then heated for 5 minutes at 37°C on a metal heat block. Pap-pen was used to draw a hydrophobic boundary around each section and let dry for 5 minutes at room temperature. Sections were incubated with 500 μL PBS to dissolve and remove the OCT embedding matrix and fixed with 500 μL of 0.5% PFA in PBS for exactly 5 minutes at room temperature. Fixation was quenched by removing the PFA and promptly adding 1.25M Glycine in PBS, one quick wash and one wash for 5 minutes at room temperature. After washing twice in PBS for 5 minutes each, the sections were permeabilized in Permeabilization buffer (0.5% TritonX100 in PBS) for 15 minutes at room temp, and then washed in PBS 3 times for 5 minutes. The sections were incubated with 0.1N HCl for 5 minutes and washed 3 times in PBS (two quick washes, one 5

minutes wash). The section were heated for 5 minutes in pre-warmed PBS at 65°C in an hybridization oven, and then snap-cooled in ice-cold PBS on ice for 10 minutes. From this point onwards all steps performed in light protected conditions. For each slide, an first RT mix (14uL) was prepared containing 2.5 μL of dNTPs (stock 10 mM each), 1.5μL of caged RT primer (BL621, 100 μM stock) in water, denatured for 5 minutes in 65C and the snap cooled on ice for >2 minutes. A second RT mix (36 μL) was prepared with 10 μL of 5x SuperScript-IV buffer, 2.5 μL SuperScript-IV enzyme, 1 μL of RNAsin, 3 μL of 100 μM TSO oligo (BL617), 5 μL of 5M Betaine, 0.5 μL ET-SSB, 2.5 μL of 100 mM DTT in nuclease free water, and added to the first mix, up to a total RT reaction volume of 50uL. Any excess PBS was removed from the slides, and 50 μL of RT mix was added directly on top of the sections, which were then covered with a 22x22 mm coverslip and incubated overnight (~16hrs) at 42°C in a sealed humid chamber.

*Barcoding:* The index oligos for the ligations were annealed at 45uM final in 2x SCC in the following pairs: BL599/BL623 (cycle 1, SZV) and BL601/BL624 (cycle 1, cortex). The annealing thermocycler conditions were as follows: 95°C for 5 minutes, ramp down to 65°C at 0.1°C /sec, 65°C for 5 minutes, ramp down to 4°C at 0.1°C /sec, hold at 4°C. The slides were retrieved from the RT reaction, washed one in PBS then twice in 2x SSC for 5 minutes each. Nuclei were stained by incubating the section with Draq5 (1:1000 dilution in 2x SSC) for 10 minutes, and then washed 2 times for 5 minutes in 2x SSC. Since the RT primer was directly caged, we proceed with two consecutive rounds of uncaging/ligation to mark the SVZ and cortex areas (bilaterally). For the uncaging, the slides were mounted with 200 μL of 2x SSC and a thin glass coverslip (22x22mm). The uncaging was performed on a SP5 Leica confocal system fitted with a 30mW 405nm solid state laser, illuminating each ROI for 5 minutes with the 405nm laser at maximum power. The slides were washed in 2x SSC, once quickly and once for 5 minutes, and then incubated for 5 minutes in 1x Ligation buffer (50 mM TrisHCl pH7.4, 10 mM $MgCl_2$, 10 mM DTT, 1mM ATP). For each sample, a ligation mix (50 μL) was prepared as follows: index oligos (3 μM, alternating BL599/BL623and BL624/BL601 as appropriate), T4 ligase (100U/ μL, NEB) in 1x T4 ligation buffer. The ligation mix was added directly to the sections, covered with a 22x22mm glass coverslip, and the reactions were incubated for 30 minutes at 25°C in a humid chamber. The slides were then washed in 2x SSC + Draq5 (1:1000) once for 10 minutes, 2x SSC + 10%EC once for 5 minutes and 2x SSC once for 5 minutes. After the serial ligations, a hybridization chamber was mounted on the slides, and the sections were lysed in 200 μL of Lysis Buffer (0.8 U/mL in RIPA buffer) at 60°C overnight (~16 hrs) in a sealed humid chamber. All the following steps were not light protected anymore.

*Library preparation:* The lysate was collected and purified with MinElute Reaction Clean-up column (Qiagen), and eluted in 25 μL of EB buffer. A small aliquot of the lysate (2 μL) was used as input for a qPCR to measure the optimal cycling conditions for the amplification of the libraries. The qPCR reaction was set up as follows: 2 μL of purified lysate, 25 μL of 2x Q5 master mix, 0.25 μL of 50 μM universal forward Truseq primer, 0.5 μL of 50 μM index A012 Truseq primer 2.5 μL of 20x EVA-Green. The cycling protocol was 98°C for 3 minutes, 40 cycles of 98°C for 15 seconds, 68°C for 20 seconds, 72°C for 20 seconds, plate reading step. The number of cycles required to reach 1/3 of the maximum signal was selected for the final amplification of the libraries. The final amplification reaction was set as follows: 10 μL of purified lysate, 25 μL of 2x Q5 master mix, 0.25 μL of 50 μM universal forward Truseq primer, 0.5 μL of 50 μM index A0XX Truseq primer, 2.5 μL of 20x EVA-Green. The cycling conditions were the same as the qPCR. The PCR reactions were purified with AmpureXP beads (0.8x) and eluted in 20 μL of EB buffer. The libraries were analyzed on a Tapestation (D5000 cartridge) and quantified with KAPA library quantification kit prior to pooling. Sequencing was performed on a Novaseq (Illumina) instrument.

### Transposome assembly

The adapters for transposome assembling were annealed at 50 μM each in 1x Annealing buffer (40 mM TrisHCl pH 8.0, 50 mM NaCl) with the following thermocycling protocol: 95°C for 5 minutes, ramp down to 65°C at 0.1°C /sec, 65°C for 5 minutes, ramp down to 4°C at 0.1°C /sec, hold at 4°C. The oligos pairs used were BL699/BL538 (BALI-adapter, universal ligation root) and BL515/BL538

(A-adapter, single modality ATAC) or BL749/BL538 (A-adapter, multi-omics). The transposomes were assembled by mixing 3 μL of annealed BALI-adapter, 3 μL of annealed A-adapter, 6 μL of unloaded Tn5 and incubating the mix at 23°C for 30 minutes and then used immediately afterwards.

**In situ ATAC**

*Tissue preparation and transposition:* Cryosections of adult brain were retrieved from cold storage and equilibrated for 5 minutes at room temperature and then heated for 5 minutes at 37°C on a metal heat block. Pap-pen was used to draw a hydrophobic boundary around each section, and let dry for 5 minutes at room temperature. Sections were incubated with 500 μL PBS to dissolve and remove the OCT embedding matrix, and fixed with 500 μL of 0.5% PFA in PBS for exactly 5 minutes at room temperature. Fixation was quenched by removing the PFA and promptly adding 1.25M Glycine in PBS, one quick wash and one wash for 5 minutes at room temperature. After washing twice in PBS for 5 minutes each, the sections were permeabilized in Permeabilization buffer (10 mM TrisHCl pH 7.4, 10 mM NaCl, 3 mM $MgCl_2$, 0.01% Tween-20, 0.01% IGEPAL, 0.001% Digitonin, 1% BSA, 10 μL murine RNase Inhibitor) for 15 minutes at room temperature. The sections were washed in 1x Wash Buffer (10 mM TrisHCl pH 7.4, 10 mM NaCl, 3 mM $MgCl_2$, 0.1% Tween-20, 1% BSA, 10 μL murine RNase Inhibitor) once quickly and once for 5 minutes, then in PBS twice for 5 minutes. For each section, 200 μL of transposition mix were prepared as follows: 2 μL of loaded Tn5 in 10 mM Tris-HCl pH 7.6, 5 mM MgCl2, 10% Dimethyl Formamide, 0.33x PBS, 0.1% Tween-20, 0.01% Digitonin. Each section was sealed with an adhesive hybridization chamber and incubated with 200 μL of tagmentation mix for 35 minutes at 37°C in a humid chamber. The slides were retrieved and washed with 40 mM EDTA once quickly, and once for 5 minutes, then 3 times in 1x PBS for 5 minutes each and 2 times in 2x SSC for 5 minutes each.

*Barcoding:* The index oligos for the ligations were annealed at 45 μM final in 1xSSC in the following pairs: BL680/BL676 (cycle 1, bulk), BL681/BL677 (cycle 2, bulk), BL683/BL642 (cycle 3, bulk), BL732/BL601 (cycle 4A, Dentate Gyrus), BL733/BL677 (cycle 4B, cortex), BL732/BL641 (cycle 1, single ligation bulk). The annealing thermocycler conditions were as follows: 95°C for 5 minutes, ramp down to 65°C at 0.1C/sec, 65°C for 5 minutes, ramp down to 4°C at 0.1°C /sec, hold at 4°C. The spatial barcodes were encoded in the tissue with the following protocol, which was conducted under light protected condition for its entirety: cycle 1 ligation, cycle 2 uncaging (bulk), cycle 2 ligation, cycle 3 uncaging (bulk), cycle 3 ligation, cycle 4A uncaging (Dentate Gyrus), cycle 4A ligation, cycle 4B uncaging (cortex), cycle 4B ligation. Bulk uncaging was performed with a UV transilluminator fitted with 365 nm bulbs for 15 minutes at room temperature, with the section covered with 500 μL of 2x SSC. Spatial uncaging was performed on a SP5 Leica confocal system fitted with a 30mW 405nm solid state laser, illuminating each ROI for 10 minutes with the 405nm laser at maximum power. Following uncaging, the slides were washed in 2x SSC, once quickly and once for 5 minutes. Ligations were performed as follows. Sections were incubated for 5 minutes in 1x Ligation buffer (50 mM TrisHCl pH7.4, 10 mM MgCl2, 10 mM DTT, 1mM ATP). For each sample, a ligation mix (50 μL) was prepared as follows: index oligos (3 μM), T4 ligase (100 U/μL, NEB) in 1x T4 ligation buffer. The ligation mix was added directly to the sections, covered with a 22x22mm glass coverslip, and the reactions were incubated for 30 minutes (45 minutes for cycle 1) at 25°C in a humid chamber. The slides were then washed in 2x SSC + Draq5 (1:1000) once for 10 minutes, 2x SSC + 10%EC once for 5 minutes and 2x SSC once for 5 minutes. After the serial ligations, an adhesive hybridization chamber was mounted on the slides, and the sections were lysed in 200 μL of Lysis Buffer (0.8 U/mL in RIPA buffer) at 60°C overnight (~16 hrs) in a sealed humid chamber.

*Library preparation:* All the following steps were not carried out under light protected conditions. The lysate was collected and purified with MinElute Reaction Clean-up column, and eluted in 25 μL of EB buffer. Libraries were amplified via quantitative PCR as follows. For each sample, a 50 μL PCR mix was prepared mixing 5 μL of purified sample, 25 μL of 2x Q5 Mastermix, 1.25 μL of 50 μM P5 primer (BL727), 1.25 μL of 50 μM indexed P7 primer (TruSeq A0XX) in nuclease free water. The reaction was incubated in a thermocycler at 72°C for 5 minutes (gap-fill), 98°C for 3 minutes, then 5 cycles at 98°C for 20 seconds, 68°C for 20 seconds, 72°C for 20 seconds. The

pre-amplified products was kept on ice, and 5 μL were used as input for qPCR to determine the optimal number of additional cycles, The qPCR reactions were set up as follows: 5 μL of the pre-amplified material, 5 μL of 2x Q5 master mix, 0.25 μL of 50 μM P5 primer (BL727), 1.25 μL of 50 μM indexed P7 primer (TruSeq A0XX), 2.5 μL of 20x EVA-Green in nuclease free water. The cycling protocol was 98°C for 3 minutes, 40 cycles of 98°C for 20 seconds, 68°C for 20 seconds, 72°C for 20 seconds with a plate reading step. The number of cycles required to reach 1/3 of the amplification curve plateau was selected for the final amplification of the libraries. The PCR reactions were purified with AmpureXP beads (0.8x) and eluted in 20 μL of EB buffer. The libraries were analyzed on a Tapestation (D5000 cartridge) and quantified with KAPA library quantification kit prior to pooling. Sequencing was performed on a Novaseq (Illumina) instrument.

**In situ multi-omics**

*Tissue preparation, transposition and RT:* All steps were performed under RNase-free conditions. Cryosections of adult brain were retrieved from cold storage and equilibrated for 5 minutes at room temperature and then heated for 5 minutes at 37°C on a metal heat block. Pap-pen was used to draw a hydrophobic boundary around each section, and let dry for 5 minutes at room temperature. Sections were incubated with 500 μL PBS to dissolve and remove the OCT embedding matrix, and fixed with 500 μL of 0.5% PFA in PBS for exactly 5 minutes at room temperature. Fixation was quenched by removing the PFA and promptly adding 1.25 M Glycine in PBS, one quick wash and one wash for 5 minutes at room temperature. After washing twice in PBS for 5 minutes each, the sections were permeabilized in Permeabilization buffer (10 mM TrisHCl pH 7.4, 10 mM NaCl, 3 mM $MgCl_2$, 0.01% Tween-20, 0.01% IGEPAL, 0.001% Digitonin, 1% BSA, 10 μL murine RNase Inhibitor) for 15 minutes at room temp. The sections were washed in 1x Wash Buffer (10 mM TrisHCl pH 7.4, 10 mM NaCl, 3 mM $MgCl_2$, 0.1% Tween-20, 1% BSA, 10 μL murine RNase Inhibitor) once quickly and once for 5 minutes, then in PBS twice for 5 minutes. For each section, 200 μL of transposition mix were prepared as follows: 2uL of loaded Tn5 in 10 mM Tris-HCl pH 7.6, 5 mM MgCl2, 10% Dimethyl Formamide, 0.33x PBS, 0.1% Tween-20, 0.01% Digitonin. Each section was sealed with an adhesive hybridization chamber and incubated with 200 μL of tagmentation mix for 35 minutes at 37°C in a humid chamber. The slides were retrieved and washed with 40 mM EDTA once quickly, and once for 5 minutes, then with PBS for 3 times for 5 minutes. The sections were heated for 5 minutes in pre-warmed PBS at 65°C in a hybridization oven, and then snap-cooled in ice-cold PBS on ice for 10 minutes. For each slide, a first RT mix (14uL) was prepared containing 2.5 μL of dNTPs (stock 10 mM each), 1.5μL of phosphorylated RT primer (BL730, 100 μM stock) in water, denatured for 5 minutes in 65°C and the snap cooled on ice for >2 minutes. A second RT mix (36 μL) was prepared with 10 μL of 5x SuperScript-IV buffer, 2.5μL SuperScript-IV enzyme, 1 μL of RNAsin, 3 μL of 100 μM TSO oligo (BL617), 5 μL of 5M Betaine, 2.5 μL of 100 mM DTT in nuclease free water, and added to the first mix, up to a total RT reaction volume of 50 μL. Any excess PBS was removed from the slides, and 50 μL of RT mix was added directly on top of the sections, which were then covered with a 22x22 mm coverslip and incubated overnight (~16hrs) at 42°C in a sealed humid chamber.

*Barcoding:* The slides were retrieved from the RT reaction, washed one in 1x PBS then twice in 2x SSC for 5 minutes each. The index oligos for the ligations were annealed at 45 μM final in 1x SSC in the following pairs: BL680/BL676 (cycle 1, bulk), BL681/BL677 (cycle 2, bulk), BL683/BL642 (cycle 3, bulk), BL732/BL601 (cycle 4A, Dentate Gyrus), BL733/BL677 (cycle 4B, CA1), BL732/BL641 (cycle 1, single ligation bulk). The annealing thermocycler conditions were as follows: 95°C for 5 minutes, ramp down to 65°C at 0.1°C /sec, 65°C for 5 minutes, ramp down to 4°C at 0.1°C /sec, hold at 4°C. The spatial barcodes were encoded in the tissue with the following protocol, which was conducted under light protected condition for its entirety: cycle 1 ligation, cycle 2 uncaging (bulk), cycle 2 ligation, cycle 3 uncaging (bulk), cycle 3 ligation, cycle 4A uncaging (Dentate Gyrus), cycle 4A ligation, cycle 4B uncaging (CA1), cycle 4B ligation. Bulk uncaging was performed with a UV transilluminator fitted with 365 nm bulbs for 15 minutes at room temperature, with the section covered with 500 μL of 2x SSC. Spatial uncaging was performed on a SP5 Leica confocal system fitted with a 30mW 405nm solid state laser, illuminating each ROI for 10 minutes

with the 405nm laser at maximum power. Following uncaging, the slides were washed in 2x SSC, once quickly and once for 5 minutes. Ligations were performed as follows. Sections were incubated for 5 minutes in 1x Ligation buffer (50 mM TrisHCl pH7.4, 10 mM $MgCl_2$, 10 mM DTT, 1mM ATP). For each sample, a ligation mix (50 μL) was prepared as follows: index oligos (3 μM, as appropriate), T4 ligase (100 U/μL, NEB) in 1x T4 ligation buffer. The ligation mix was added directly to the sections, covered with a 22x22mm glass coverslip, and the reactions were incubated for 30 minutes (45 minutes for cycle 1) at 25°C in a humid chamber. The slides were then washed in 2x SSC + Draq5 (1:1000) once for 10 minutes, 2x SSC + 10%EC once for 5 minutes and 2x SSC once for 5 minutes. After the serial ligations, an adhesive hybridization chamber was mounted on the slides, and the sections were lysed in 200 μL of Lysis Buffer (0.8 U/mL in RIPA buffer) at 60°C overnight (~16 hrs) in a sealed humid chamber.

*Lysis and DNA-RNA library separation:* All the following steps were not carried out under light protected conditions. The lysate (200 μL) was collected and an aliquot (150 μL) was treated with 10 μL of 100 mM PMSF for 10 minutes at room temperature to inactivate the proteinase K prior to biotin pulldown to separate the Tn5 fragments (biotinylated) from cDNA. For each sample, 10uL of MyOne C1 beads were washes 3 times in 1xB&W-T buffer, and then resuspended in 150 μL of 2xB&W-T buffer. The lysate was then added and incubated with the beads for 60 minutes at room temperature. After this Incubation we collected the supernatant as our cDNA enriched fraction for RNA libraries, while the beads represented our Tn5 fragments enriched fraction for ATAC libraries.

*Library preparation – DNA:* The beads were washed 3 times in 1xB&W-T buffer for 5 minutes, once in 1xST buffer for 5 minutes and then directly used as input for library amplification via precise PCR as follows. For each sample, a 50 μL PCR mix was prepared mixing 25 μL of 2x Q5 Mastermix, 2.5 μL of 10 μM P5 primer (BL727), 2.5 μL of 10 μM indexed P7 primer (TruSeq A0XX) in nuclease free water, and adding it directly to the washed bead pellet. The reaction was incubated in a thermocycler at 72°C for 5 minutes (gap-fill), 98°C for 3 minutes, then 5 cycles at 98°C for 20 seconds, 68°C for 20 seconds, 72°C for 20 seconds. The pre-amplified products was kept on ice, and 5 μL were used as input for qPCR to determine the optimal number of additional cycles, The qPCR reactions were set up as follows: 5 μL of the pre-amplified material (after removal of the beads), 5 μL of 2x Q5 master mix, 0.5 μL of 10 μM P5 primer (BL727), 0.5 μL of 10 μM indexed P7 primer (TruSeq A0XX), 2.5 μL of 20x EVA-Green in nuclease free water. The cycling protocol was 98°C for 3 minutes, 40 cycles of 98°C for 20 seconds, 68°C for 20 seconds, 72°C for 20 seconds with a plate reading step. The number of cycles required to reach 1/3 of the amplification curve plateau was selected for the final amplification of the libraries. The PCR reactions were purified with AmpureXP beads (1.2x) and eluted in 20 μL of EB buffer. The libraries were analyzed on a Tapestation (D5000 cartridge) and quantified with KAPA library quantification kit prior to pooling. Sequencing was performed on a Novaseq (Illumina) instrument.

*Library preparation – RNA:* Separately, the cDNA fraction was purified using Monarch Reaction clean-up columns and eluted in 12.5uL. The cDNA was treated with RNaseH by adding 1.5 μL of 10x RNaseH buffer and 1 μL RNaseH enzyme to each sample, and then incubating the mix at 37°C for 30 minutes, 65°C for 20 minutes. The cDNA was amplified to via precise PCR as follows. For each sample, a 50 μL PCR mix was prepared mixing 15 μL of RNaseH treated sample, 25 μL of 2x Q5 Mastermix, 2.5 μL of 10 μM forward primer (BL753), 2.5 μL of 10 μM reverse primer (BL752) in nuclease free water. The reaction was incubated in a thermocycler at 98°C for 3 minutes, then 5 cycles at 98°C for 15 seconds, 67°C for 20 seconds, 72°C for 60 seconds. The pre-amplified products was kept on ice, and 5 μL were used as input for qPCR to determine the optimal number of additional cycles, The qPCR reactions were set up as follows: 5 μL of the pre-amplified material, 5 μL of 2x Q5 master mix, 0.5 μL of 10 μM forward primer (BL753), 0.5 μL of 10 μM reverse primer (BL752), 2.5 μL of 20x EVA-Green in nuclease free water. The cycling protocol was 98°C for 3 minutes, 40 cycles of 98°C for 15 seconds, 67°C for 20 seconds, 72°C for 60 seconds with a plate reading step. The number of cycles required to reach 1/3 of the amplification curve plateau was selected for the final amplification of the libraries. The PCR reactions were purified with AmpureXP beads (0.8x) and eluted in 20 μL of EB buffer. A small aliquot (2 μL) was analyzed on a Tapestation D5000 screentape. The amplified cDNA fraction was then tagmented with Tn5 loaded with a single

adapter (BL371/BL515) in a reaction set up as follows: 50 ng of pre-amplified cDNA, 0.5 μL loaded Tn5 enzyme in 10 mM Tris-HCl pH 7.6, 5 mM $MgCl_2$, 10% Dimethyl Formamide, 0.33x PBS, 0.1% Tween-20, 0.01% Digitonin in 50uL final. The tagmentation reaction was incubated at 37°C for 30 minutes, and inactivated adding 50 μL of 80 mM EDTA and 2.5 μL of 2% SDS solution for 15 minutes at room temperature. The reactions were purified were purified with AmpureXP beads (0.8x) and eluted in 20 μL of EB buffer. A small aliquot (2 μL) was analyzed on a Tapestation. The purified reaction was amplified to generate ATAC libraries as follows: 18 μL of Tn5-cDNA, 2.5 μL of 10uM P5 primer (BL727), 2.5 μL of 10 μM indexed P7 primer (Ad2.X) in nuclease free water. The cycling protocol was 72°C for 5 minutes (gap-fill), then 98°C for 1 minutes and 5 cycles of 98°C for 10 seconds, 68°C for 20 seconds, 72°C for 45 seconds, with a final extension at 72°C for 5 minutes. The PCR reactions were purified with AmpureXP beads (0.8x) and eluted in 20 μL of EB buffer. The libraries were analyzed on a Tapestation (D5000 screentape) and quantified with KAPA library quantification kit prior to pooling. Sequencing was performed on a Novaseq (Illumina) instrument.

### BALI barcode demultiplexing

For all sequencing experiments including BALI barcodes (regardless of length), the fastq files produced by high-throughput sequencing were processed through the following pipeline using custom bash shell scripts: 1) UMI extraction and annotation using the umitools package (v0.5.5) with the command *umi_tools extract–-bc-pattern=NNNNNN*, 2) extraction of reads including each first (spatial) BALI barcode from both paired fastq files using the *cutadapt* package (v4.1) (options *-g ^[barcode], -e 1, --pair-filter-both, --match-read-wildcards*), 3) Extraction of successive BALI barcodes from the output of step 2, using the same command and options, 4) Extraction of the final adapter placed before the sequencing library itself (RT oligo for RNAseq, tn5 mosaic end for ATAC-seq) using the same command as above. The strategy was performed iteratively to produce a pair of read1/read2 fastq files for each complete BALI barcode, as well as to produce fastq files for "partial" barcodes caused by incomplete ligations (i.e. in cases in which position 1 of a barcode ligated to the overhang-compatible position 4 rather than to position 2).

Further analysis was performed on all reads featuring full barcodes, as well as on those featuring a complete barcode fragment including position 4 (the spatially defined one) as well as position 3, plus the end adapter (RT or mosaic end). These reads corresponded to molecules that could be reliably assigned to a specific region.

### BALI RNA sequencing analysis

The read 1 fastq file from each spatial region was mapped on the mm10 reference genome (source: Illumina igenomes) using the STAR package (v 2.7.9a) with the following options: --*outReadsUnmapped Fastx –outSAMtype BAM Unsorted–-quantmode GeneCounts*. The resulting BAM files were then sorted and indexed using the *samtools* package (v1.9) commands. To produce UMI counts for each gene (as opposed to the raw counts produced by STAR), each BAM file was run through the *featureCounts* command from the *Subread* package (v2.0.3) with default options against the same mm10 genome annotation described above. The resulting annotated BAM file was sorted again using samtools sort. UMIs counts per gene were calculated using the *umi_tools count* command (from the umitools package v0.5.5) with options*–-per-gene–-gene-tag=XT–-assigned-status-tag=XS,* and UMIs were deduplicated using the *umi_tools group* command (to produce an overall quantification of all UMIs in each region) and *umi_tools dedup* command (to produce a deduplicated BAM file). The deduplicated BAm files were sorted and indexed a third time, and genome coverage was calculated using the *bamCoverage* command from the *deeptools* package (v3.5.1) with the following options*–-normalizeUsing RPKM–-binSize 1.*

For the RNA only experiment on e16.5 mouse embryos, differential expression was performed by merging the UMI count matrices produced by the procedure described above. Each count was normalized by dividing it by the total number of gene-assigned UMIs in the library and multiplying

it by 1,000,000, then fold change and log10 change ratios between the SVZ and the cortical areas were calculated using excel. For the multi-omic experiment on adult mouse hippocampus, the UMI count matrices from each area and replicate were merged and normalized as above, and the fold and log10 change ratios calculated using the average expression across replicates.

### Laser capture microdissection data analysis

Raw paired-end sequencing files from each LCM-dissected area were first filtered using the cutadapt package (v4.1) to remove the Illumina sequencing adapters from both the 5' and 3' of each sequencing read. The filtered read 1 was then mapped on the mm10 was mapped on the mm10 reference genome (source: Illumina igenomes) using the STAR package (v 2.7.9a) with the following options: *--outReadsUnmapped Fastx –outSAMtype BAM Unsorted --quantmode GeneCounts*. The resulting BAM files were then sorted and indexed using the *samtools* package (v1.9) commands. Genome coverage was calculated using the *bamCoverage* command from the *deeptools* package (v3.5.1) with the following options *--normalizeUsing RPKM --binSize 1.* Differential expression was performed by merging the count matrices produced by the procedure described above. Each count was normalized by dividing it by the total number of gene-assigned reads in the library and multiplying it by 1,000,000 then fold change and log10 change ratios between the SVZ and the cortical areas were calculated using excel.

### Re-analysis of existing literature data for 10X Visium

The raw data from Vanrobaeys *et al*. was obtained from GEO accession GSE223066. The spaceranger output matrices for samples *Visium-HC5* to *Visium-HC9* (corresponding to "home cage" control conditions) were downloaded and processed using the *scanpy* package (v1.9.8) under python. First basic statistics, including percent of reads mapping to mitochondrial genes and total UMIs, were calculated for each spot. Second, spots with <5000 or >35000 UMIs, or with more than 20% Mt reads, were excluded. A custom script (using the *holoviews* and *bokeh* packages) was then used to define, on the high-resolution H&E image associated with each Visium dataset, areas matching the two regions profiled by the BALI multi-omic experiment. The UMI counts from all the spots included in each of the areas were then summed (pseudobulking), and the resulting count matrices for each replicate dataset (HC5 to HC9) were merged together as a single dataset.

Differential expression was performed as follows: first each count value was normalized by dividing it by the total number of UMIs in that sample/area and multiplying it by 1,000,000 then fold change and log10 change ratios between the DG and CA1 areas were calculated on the average of the UMIs across all replicates for each areas. The fold and log ratios obtained using this method were compared to those obtained by performing normalization and differential expression testing using the R *DeSeq2* package, and showed similar results

### Gene enrichment visualization plots

Gene enrichments were visualized using a custom python script using the following modules: *numpy, holoviews, bokeh*. The script comprised the following sterps: 1) an histological image corresponding to the anatomical areas being measured was used to define two representative polygons corresponding to the profiled areas, 2) the log10 change value for a gene to be visualized was divided by a user-defined value set as maximum color intensity, 3) the resulting value was used to set the color intensity for the polygon in which the gene was upregulated, 4) the two color-graded polygons were drawn overlaid to the anatomical regions of interest. The minimum and maximum scaling values used were -1 and +1 log10 units for LCM data and BALI data on e16.5 embryos, and -0.5 and +0.5 log10 units for multi-omic BALI data and the matching Visium data from *Vanrobaeys et al.*

## ATAC-Seq sequencing analysis

Both read 1 and read 2 files from each spatial region were mapped to the mm10 reference genome (source: Illumina igenomes) using the *bowtie2* package (v2.5.1) using the following options: *--local --very-sensitive-local --soft-clipped-unmapped-tlen --dovetail --no-mixed -q --no-discordant --phred33 -I 10 -X 1000*. The resulting SAM files were converted to BAM, sorted and indexed using the *samtools* package (v1.9). Genome coverage was calculated using the *bamCoverage* command from the *deeptools* package (v3.5.1) with the following options *-normalizeUsing None --binSize 3 --scaleFactor [scale_factor]*. The scale factor was calculated as *1 / total mapped reads* for each library. Peaks were identified using the macs2 package (v2.2.9.1) using the following options *-f BAMPE --nomodel --keep-dup=all.*

## DNA motif enrichment

DNA motif enrichment was performed using the HOMER tool (v5.1) with the *findMotifsGenome* command, using the *mm10* genome release as reference and the DNA binding factors in the *all.motifs* file included in the default installation as motif database. The following options were used: *-size 200 -S 20 -len 8,10* (de novo motif finding limited to 8 and 10 nt motifs). The analysis was performed on the peak summits generated from the *macs2* command above, using a 200nt region surrounding the summits as substrate. For the multi-omic experiment, the HOMER analysis was performed separately on the peak results obtained from the dentate gyrus-specific and CA1-specific reads (after barcode demultiplexing). Motifs that were enriched on just one of the libraries were marked as region-specific, and motifs that were enriched in both were marked as common.

## Re-analysis of existing literature data for integrated scRNAseq/scATACseq

Vanrobaeys *et al.*[47] includes matched single-cell RNA-seq (produced using the split-seq protocol from Parse Biosciences) and single-cell ATAC-seq (produced using the 10X ATAC-seq protocol) for the hippocampus of control mice (home cage condition). We used this data to identify cellular populations corresponding to the dentate gyrus granule cells and CA1 pyramidal cells, and match their genome coverage profile to the one obtained by BALI in the corresponding areas.

All analysis was performed in R using packages *AnnotationHub (v3.6.0), Seurat (v5.0.1) and Signac (v1.12.9004)*. Annotation data was extracted from the EnsemblDB Mus Musculus annotation v98 (corresponding to the annotation used by the cellranger-ATAC reference genome used in the paper).

scRNAseq analysis and scATACseq analysis were performed as described in the “Split-Seq Analysis” and “Single Nuclei ATAC-Sequencing data analysis” sections of Vanrobaeys *et al*, including integration between the two datasets. The outputs from the cellranger-ATAC pipeline and split-pipe pipeline were downloaded from GEO accession GSE223066. For ATAC-Seq, the two replicate datasets were merged before dimensionality reduction and clustering.

The CA1 and DG populations were detected in the scRNAseq data by observing overexpression of several markers including *C1ql2, Neurod2, Synpr, Prox1* (DG) and *Mpped1* and *Neurod6* (CA1 pyramidal). Genome coverage plots were obtained for the clusters in the scATACSeq data for which the predicted transfer label from scRNAseq matched the CA1 and DG populations using the Signac *CoveragePlot* function.

## Automated BALI instrument (LightScribe)

The automated BALI instrument (from now on “LightScribe”) is a custom developed microscope with onboard fluidic subsystem based on a commercial DLP projector (InVision Firebird) provided by InVision Technologies AG (Vienna, Austria), originally developed for stereolitography 3D printing. The projector is based on a Texas Instrument DLP9000 chip (2560 x 1600 pixels) and is equipped with a high-power 405nm LED light source capable of delivering shaped illumination with

a maximum power of ~40W/cm2. The version that we used for our instrument was equipped with a projection lens (cat.name “Shindig”) capable of illuminating a field of view of approximately 3x5mm with a pixel resolution of 2um/pixel.

The LightScribe instrument has three components: 1) the projection subsystem already described 2) the microscopy subsystem, and 3) the fluidic subsystem. The three subsystems are arranged around a custom-designed vertical flow-cell holding the sample on a 3-axis motorized stage (Thorlabs). The flow cell is built from two CNC-machined aluminium units that are clamped around a standard glass slide (25x75mm, 1mm thick) holding the sample. The sample is first surrounded by a 900um thick circular silicon gasket (20mm diameter, Grace Biolabs) with one side cut off, and then installed in the flow cell which compresses the gasket creating a fluid barrier. The flow cell is opened on one side, allowing new fluid to be applied to the sample, and has a waste port on the other side, allowing the previous fluid to be drained. The assembly is mounted on the stage so that the open side faces upwards and the waste port downwards. In this configuration, buffers can be added to the slide from the top using a fine pipette or a needle, and air bubbles that form rise to the top and dissipate. The flow cell is fixed to a motorized XYZ stage that enables lateral movement for sample exploration and axial movement to focus the projector light on the sample surface through the glass slide. The microscopy subsystem is placed on the opposite side of the flow cell from the projector, and used to image the sample through a clear plastic coverslip affixed to the rear of the flow cell and in contact with the silicon gasket. The subsystem is composed as follows: 1) an excitation axis formed by a Lumencor SOLA white light engine, providing light trough a 3mm light guide, an epifluorescence light collimator (Thorlabs WFA2001), and a motorised filter wheel (Thorlabs FW102C) with filters centerd at 405nm, 488nm, 560nm, and 647nm. 2) a multi-band dichroic filter (Chroma technologies, zt405-473-559-635-748rpc) with reflection bands centerd on the same wavelengths as above, and 3) an emission axis formed by a second motorised filter wheel (Thorlabs FW120C) with filters centerd at 450nm, 520nm, 600nm and 680nm, a 0.75X tube lens (Thorlabs WFA4101), and a 12.22MP CMOS monochrome camera (Allied Vision Alvium 1800 U-511m). Imaging is performed using a 4X plan-achromat objective (Olympus RMS4X) mounted on a linear motorized stage allowing focusing on the sample. Finally, the fluidics subsystem is mounted on top of the flow cell, and is formed by a water-cooled plate (Thorlabs WPC4X6) fitted with a steel 1.5ml Eppendorf tube holder and connected to a circulating refrigerated water bath held at 4C, a 3-axis robotic arm (Rotrics DexArm with pen attachment), and a peristaltic pump (Gilson Minipuls 3) connected via a 1/4-28 flangeless compression fitting and a 1/4-28 luer lock adapter to a 23G needle. The robotic arm acts as a pipetting device, moving the needle inside the source buffer tubes, aspirating fluid, and dispensing it into the flow cell. Additional buffer reservoirs for wash buffers (SSC and SSC/ethylene carbonate), ligation buffer and nuclear staining buffer are placed alongside the cooled plate, either kept at room temperature (ligation and staining buffer) or at 42C on top of a thermoblock (wash buffers). Finally, a second peristaltic pump (Gilson Minipuls Evolution) is connected to the waste port via a blunt 18G needle glued to the flow cell and a luer-lock connector, allowing clearance of buffers. The entire system is connected to a windows PC via a combination of USB ports and USB to serial adapters, and controlled via a custom software built in python. The software allows basic microscope control (illumination, filters, stage movement), imaging, fluidic control (robot movement and control, pump control), and projector control (definition of the uncaging mask and of the intensity of illumination). The software includes a GUI built on top of the holoviews and panel python modules. Using the software, the user performs the following operations: 1) aligns the projection and imaging subsystems by defining the position of 4 points illuminated by the projector in the microscope image, thus ensuring that uncaging masks can be defined based on the tissue microscope image, 2) images the tissue, 3) defines regions of interest to barcode, and optionally a regular grid with a defined cell size and spacing within each region (single-cell barcoding based on cell segmentation is also possible at this stage), 4) defines the reference points for the fluidic system (location of buffer reservoirs, location of flow cell), and 5) launches an automated imaging/barcoding program defined by a csv file using a set of instructions. The microscope then performs the uncaging, wash, and ligation steps to produce the BALI barcodes on the tissue.

## Acrylamide slide preparation and coating with BALI root oligos

Before initiating automated barcode writing, slides were functionalized by adhering to them a thin polyacrylamide gel exposing streptavidin molecules, and bound to a carpet of biotin-modified root oligonucleotides capable of being ligated to BALI indices. Glass slides were first coated with 0.05% v/v BIND-silane (3-(Trimethoxysilyl)propyl methacrylate - Sigma M6514) diluted in 80% EtOH / 2% Acetic acid for 1h at room temperature, and washed in Ethanol before being air-dried. Two strips of scotch tape were used at the beginning and end of the slide to create spacers allowing the formation of a ~50 um thick gel. A solution of 4% 19:1 acrylamide-bisacrylamide (Bio-Rad 1610144), in 50 mM Tris-HCl pH 8 / 300 mM NaCl was degassed under vacuum and polymerization was initiated by addition of 0.1% ammonium persulfate and 0.1% N,N,N′,N′ - Tetramethylethylenediamine (TEMED). 160 μL of the polymerizing acrylamide solution were quickly mixed with 40ul of 2mg/ml streptavidin-acrylamide (Thermo Scientific S21379) diluted in 1X PBS, and 100 μL each were pipetted in the center of two glass plate pre-treated with gel-slick solution (Lonza 50640). The bind-silane activated slides were inverted on the drop of acrylamide solution and left to polymerize for 1h before being lifted with a razor blade. A thin acrylamide gel was left on the slide surface. Acrylamide slides were washed twice in 1X PBS, then 20 μL of a 10 μM solution of BL-940 oligonucleotide (biotin-modified BALI root) were pipetted (in a dark room) in the center of the gel, covered with a 12mm round coverslip, and incubated for 30 minutes in the dark in a humid chamber. Slides were then washed twice for 5 minutes each in 2X SSC and stored in SSC until required for the barcode writing experiment.

## Automated fluorescent barcode writing (256 areas)

Before the experiment, BALI index oligonucleotides were annealed (in the dark) according to the table below. Each oligonucleotide pair was mixed to a final concentration of 50 μM in 1X SSC, placed in a thermocycler, and annealed using the following program: 1) 95°C for 5 minutes, 2) ramp down to 65°C at 0.1°C/second, 3) hold at 65°C for 5 minutes, 4) ramp down to 12°C at 0.1°C/second, 5) hold at 12°C. Annealed indices were stored at 4°C until use. The oligonucleotide pairs were as follows: 1) ligation 1a/3a: BL646/641, 2) ligation 1b/3b: BL645/641, 3) ligation 2a/4a: BL647/643, 4) ligation 2b/4b: BL648/644, 5) final ligation: BL732/641. A polyacrylamide slide coated with a photocaged BALI root oligo was removed from SSC and installed into the flow cell of the LightScribe instrument in the dark (using a red-filtered light). The following wash buffers were placed in room temperature reservoirs on the instrument: 1) 2X SSC, 2) 2X SSC + 10% Ethylene Carbonate, 3) 1X T4 DNA ligase buffer (New England Biolabs). The flow cell was filled with 1X ligase buffer using a syringe and a needle, and the root oligo (Atto488 conjugated) was visualized using the microscope subsystem. 256 areas were defined in a regular grid with each square approximately 150 μm in size (with a 10 μm gap), and uncaging masks were defined for each ligation reaction (1a, 1b … 8a, 8b) so that each area was assigned a unique barcode with 8 bits (positions) and 2 alternative indices per position (termed barcode “4” and “5”, each labelled with a different fluorophore, Cy5 or Atto565N). Ligation mixes were prepared for each ligation reaction (1a,1b…4a,4b) by combining 30 μL of T4 DNA ligase reaction buffer, 15 μL of T4 DNA ligase (New England Biolabs M0202M), 18 μL of annealed index oligonucleotides and 237 μL nuclease-free water. The ligation mixes were transferred to 1.5ml tubes, and moved to the refrigerated holder in the instrument. For each cycle, the following steps were automatically followed: 1) 30 minutes ligation in each ligation mix, 2) 5 minutes wash in 2X SSC, 3) 5 minutes wash in 2X SSC/EC, 4) 5 minutes wash in 2X SSC, 5) 2 minutes wash in T4 ligase buffer, 6) Imaging in the Atto565N and Cy5 excitation/emission bands (roughly 800ms exposure). The images from each cycle were stacked, intensity-adjusted to correct for the decrease in signal observed after each cycle, and used to produce the figures shown in the manuscript.

For signal uniformity measurements, post-ligation fluorescence images for the signal corresponding to index b (ATTO 565) were used. A 20x20 pixel square was defined in the center of each barcoded region, and the average fluorescence intensity was measured for each of the regions positive for ATTO 565 signal in each of the cycles of barcoding. These values were averaged for all regions and used to produce the violin plot in figure 5c.

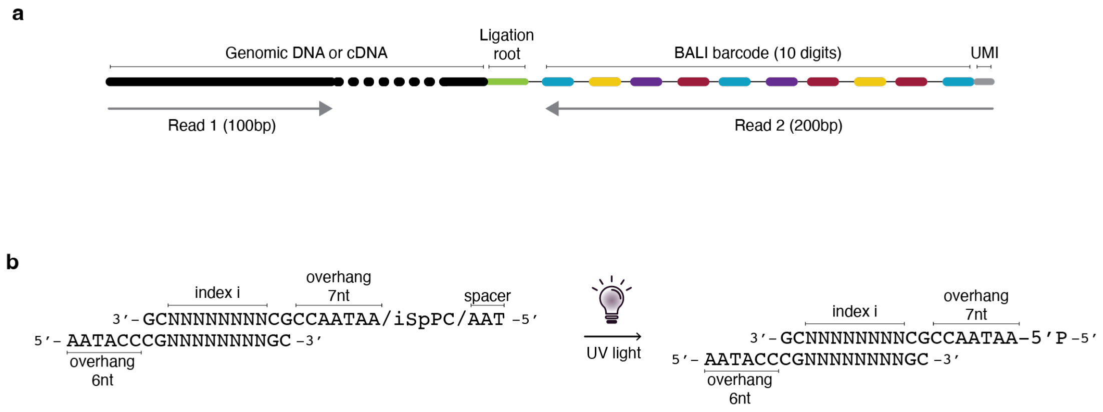


**Supplementary Figure 1. BALI library and index structure**

a) *Library structure*. Genomic or 3'end terminal of the cDNA are shown in black (dotted region to represent variable fragment sizes). The ligation root is represented in green. The BALI spatial barcode is exemplified as a 10-digit barcode, where each digit is shown as different color box. A region with the Unique Molecular Identifier is shown as a grey box. Paired end sequencing is used to read the cDNA/genomic fragment sequence (Read 1, 100 bp) and the spatial barcode plus UMI (Read 2, 200 bp). b) *Index structure.* Each index cassette is composed of two staggered complementary oligonucleotides. On the extending strand (upper) there is a variable sequence that corresponds to each individual index value, flanked by GC staples to ensure strong annealing at the ligation site. At the 5'end there is a ligation overhang (alternating between a fixed 7nt or 6nt sequence), a photo-cleavable spacer, and a terminal 5' spacer (5'-AAT-3'). On the opposite strand, there is the reverse complemented sequence to the index and staples, plus the reverse complement sequence for the orthogonal overhang (6-nt if 7nt on the leading strand, or the opposite). Upon illumination with UV light, the photo-cleavable spacer is released to leave a 5' phosphate available for T4 DNA ligase mediated ligation.

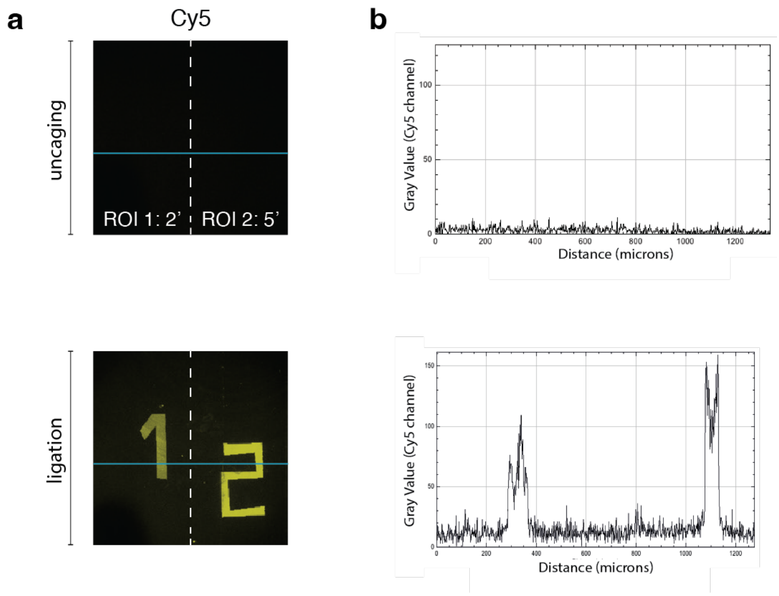


**Supplementary Figure 2. Additional results on barcode writing on solid surface with confocal microscope.**

A slide was covalently functionalized with oligos featuring a 5' terminal Cy3 fluorophore attached through an internal photo-cleavable linker. Upon UV illumination of a region of interest for 2 minutes (ROI1) or 5 minutes (ROI2), using a confocal microscope equipped with a 405nm laser, the photo-cage was released producing a 5' terminal phosphate group, and the oligo thus made available for ligation with a staggered dsDNA cassette featuring a 5' terminal Cy5. The images show the signal of the ligated cassette (Cy5), post UV illumination and post ligation (top and bottom rows, respectively). Fluorescence intensity post-ligation correlates with uncaging time, indicating gradual deprotection of the root oligo installed on the slide. Fluorescence intensity profiles along the blue horizontal line are shown on the right for each panel. Images are in false colors and color-blind friendly palette

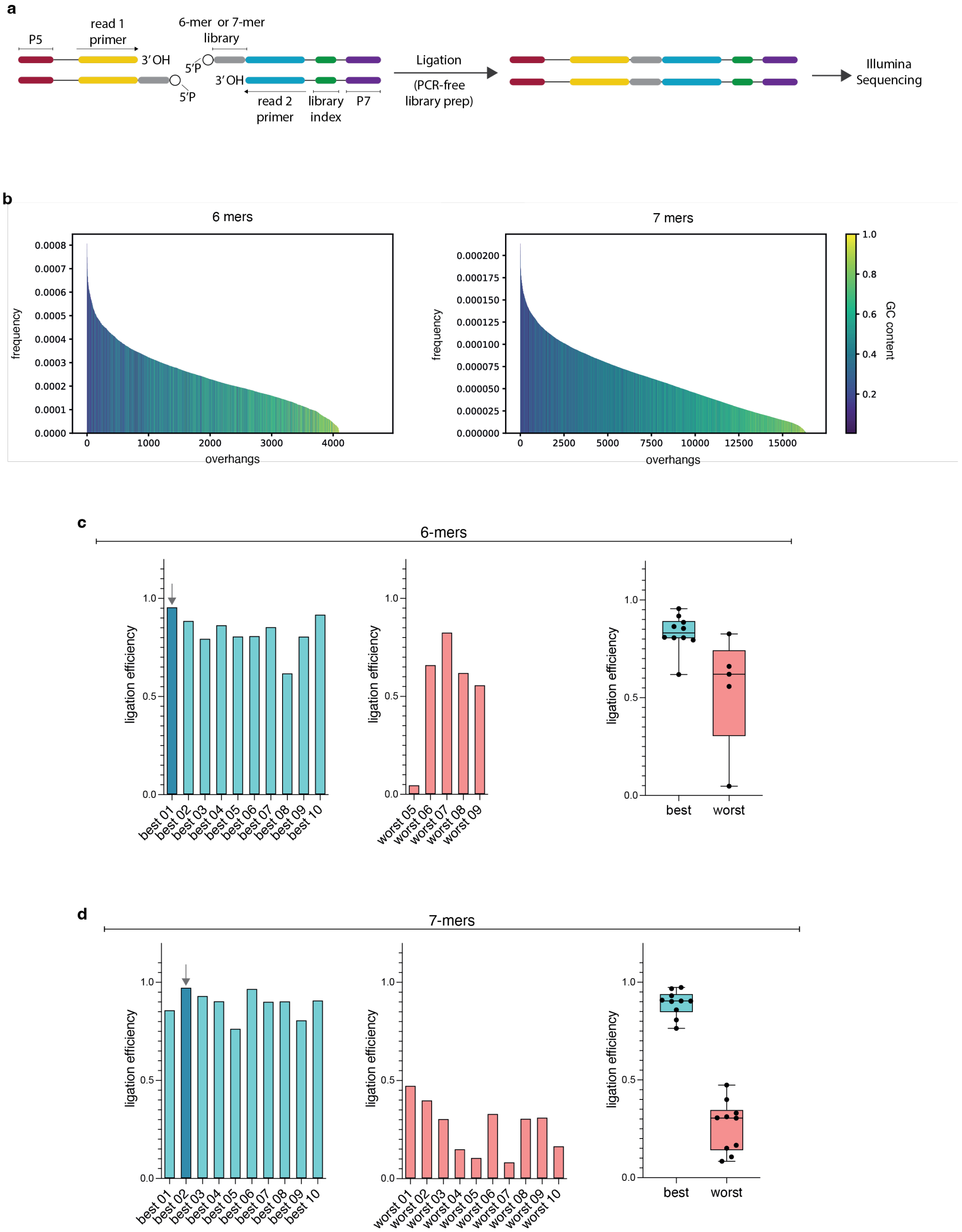
a
P5
read 1
primer
3′OH
5′P
6-mer or 7-mer
library
5′P
3′OH
read 2
primer
library
index
P7
Ligation
(PCR-free
library prep)
Illumina
Sequencing
b
6 mers
7 mers
frequency
overhangs
GC content
c
6-mers
ligation efficiency
best 01
best 02
best 03
best 04
best 05
best 06
best 07
best 08
best 09
best 10
worst 05
worst 06
worst 07
worst 08
worst 09
best
worst
d
7-mers
ligation efficiency
worst 01
worst 02
worst 03
worst 04
worst 05
worst 06
worst 07
worst 08
worst 09
worst 10

**Supplementary Figure 3. Overhang screen and validation**

a) *Experimental set-up.* A library of oligos with variable 5' ligation overhangs and terminal Illumina P5 and P7 sequences were annealed to produce double-strand DNA indices and ligated as illustrated in figure. The resulting molecules were directly sequenced through Illumina sequencing. Overhangs with different lengths, 6 or 7 nucleotides, where processed separately. b) *Bar plots representing the frequency of reads for each overhang over the total number of reads.* Overhangs were ranked by frequency (highest to lowest from left to right). The color of the bars represents the GC content of the overhang. Average across 3 replicates shown. Error bars not shown for ease of representation. c, d). *In situ validation of the efficiency validation for the best and worst overhangs identified in the screen for 6-mers (c) and 7mers (d).* The efficiency ligation was measured by densitometry on a denaturing Urea PAGE gel. The individual efficiencies are shown as bar plots, the average efficiencies are shown as box plots (central line for average, whiskers to show min and max values, box extends from 25$^{th}$ to 75$^{th}$ percentile). Blue for 'best' candidates, red for 'worst' candidates. The chosen overhangs are in darker blue and labelled with an arrow in the bar plot.

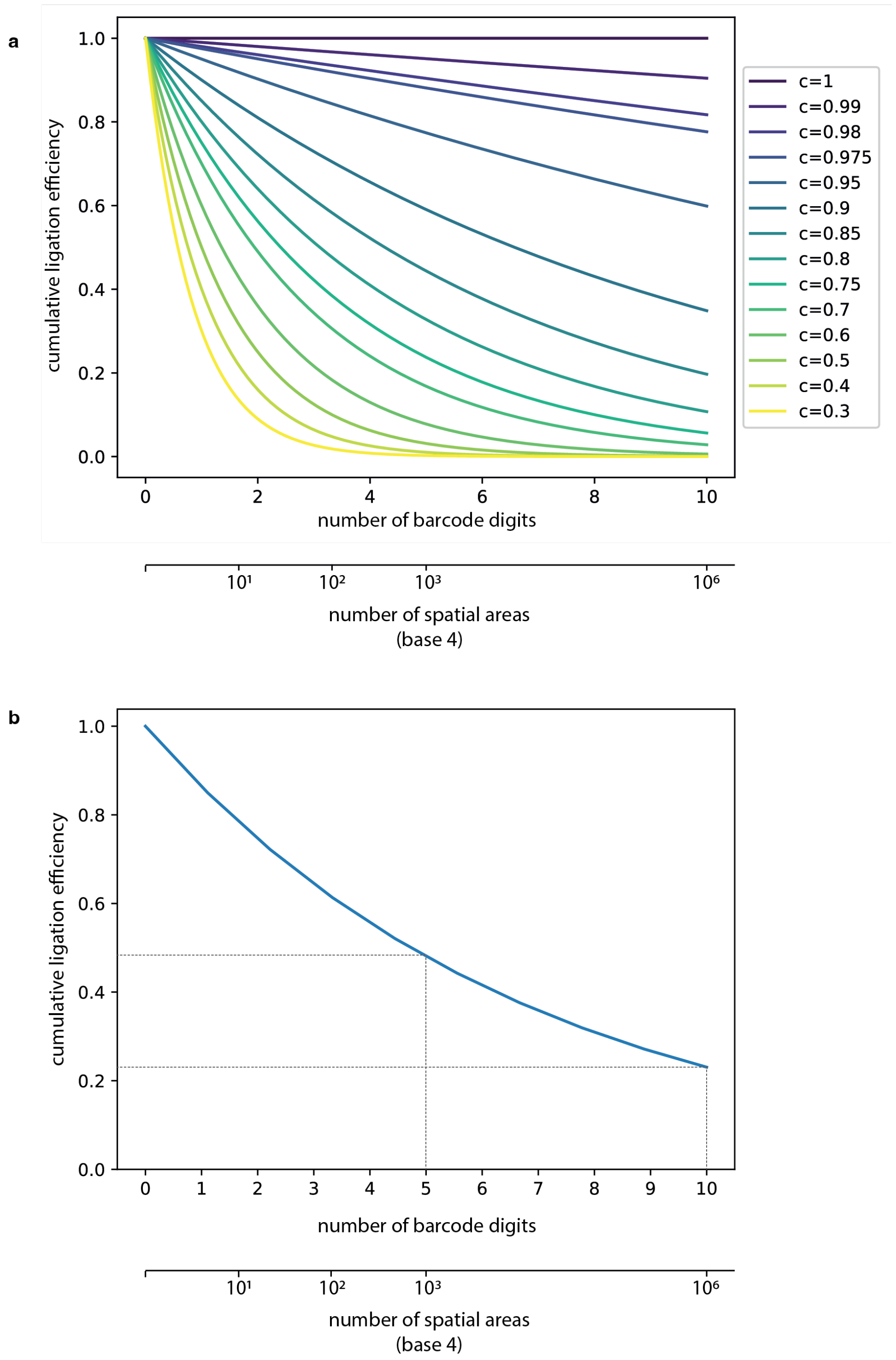
a
b
cumulative ligation efficiency
number of barcode digits
number of spatial areas
(base 4)
$10^1$
$10^2$
$10^3$
$10^6$
c=1
c=0.99
c=0.98
c=0.975
c=0.95
c=0.9
c=0.85
c=0.8
c=0.75
c=0.7
c=0.6
c=0.5
c=0.4
c=0.3

**Supplementary Figure 4. Projections for the cumulative efficiency of ligation for multi-digit barcodes.**

a) Projection of the cumulative efficiency of ligation after *n* sequential cycles depending on the average efficiency per cycle (c value = 0.3÷1). The double x-axis represents the number of cycles (*n*), and the corresponding maximum number of spatial barcodes with *n* digits and 4 different values available at each digit (base 4). b) Projection of the cumulative efficiency of after n sequential cycles based on the average efficiency per cycle measured in tissue, c=0.8635 (Fig. 2c). The double x-axis represents the number of cycles (*n*), and the corresponding maximum number of spatial barcodes with *n* digits and 4 different values available at each digit (base 4). The intercepts represent the projected cumulative efficiencies requires to encode ~ 1000 spatial areas (5 digits, base 4) or ~1 million (10 digits, base 4).

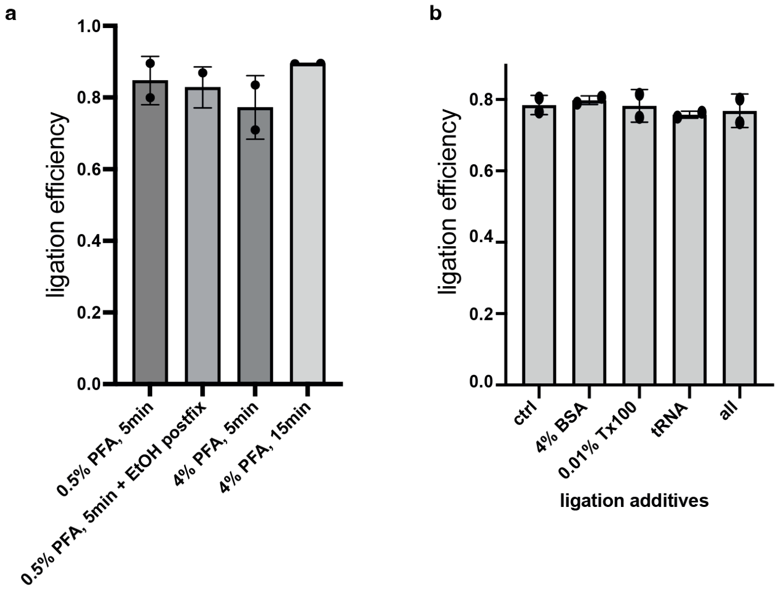


**Supplementary Figure 5. Effect of different fixation conditions and additives on ligation efficiency on tissue.** a) bar chart indicating the ligation efficiency obtained when performing ligation, in duplicate, on tissue sections fixed for different times (5 vs 15 minutes) in difference concentrations of paraformaldehyde (0.5% vs 4%). For the lower paraformaldehyde amount, an additional post-fixation in Ethanol was also tested. b) bar chart indicating the ligation efficiency obtained when performing ligation, on tissues fixed for 5 minutes in 0.5% PFA, with different additives added to the ligation mix. In all cases experiments were performed in duplicate. Error bars are standard deviation.

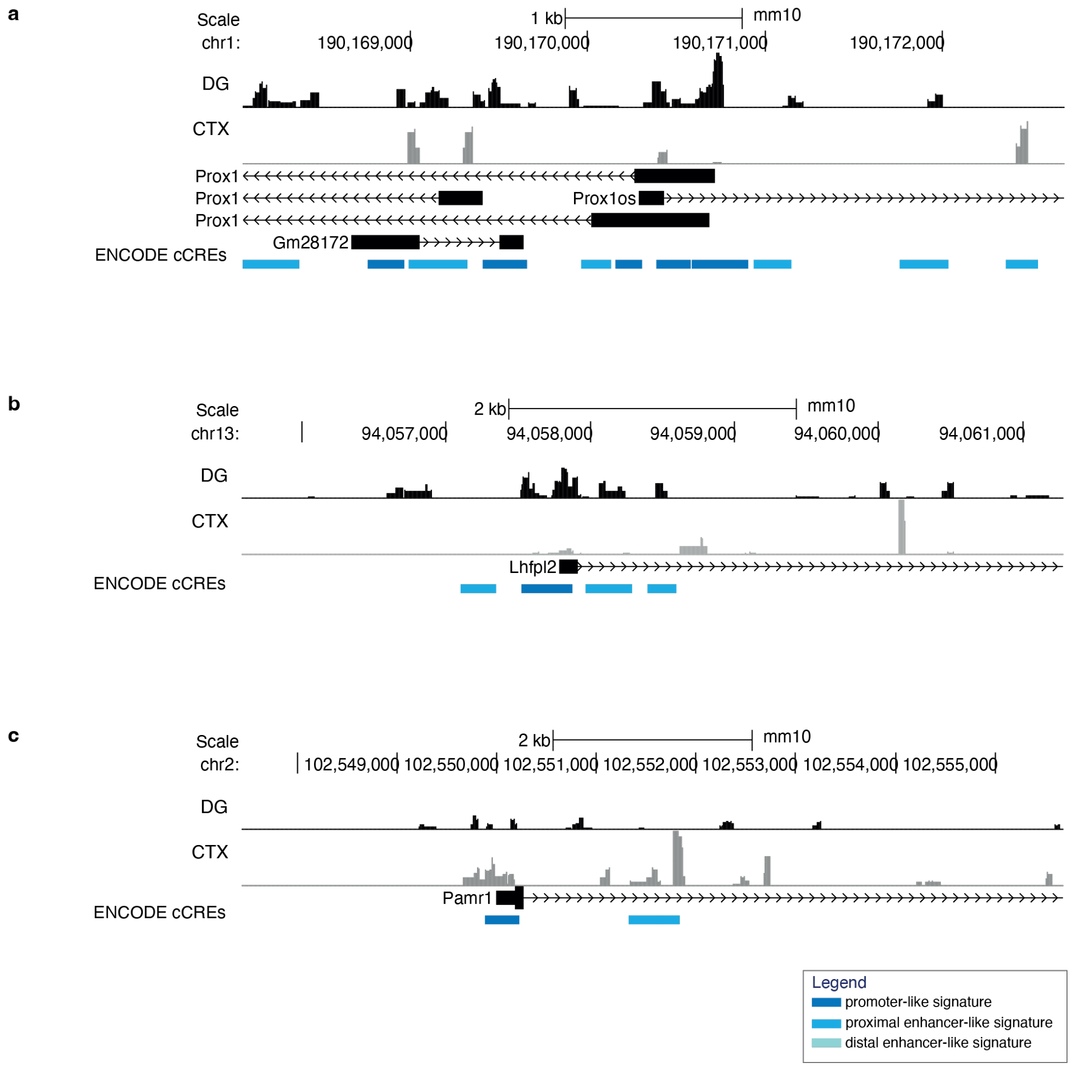


**Supplementary Figure 6. Coverage plots for chromatin accessibility at additional TSSs.** Accessibility for the DG and CTX are shown separately in black and grey, respectively. The RefSeq annotation is shown below, as well the annotation for regulatory elements from the ENCODE's cCREs database[46] a) Prox1, b) Lhfpl2, c) Pamr1

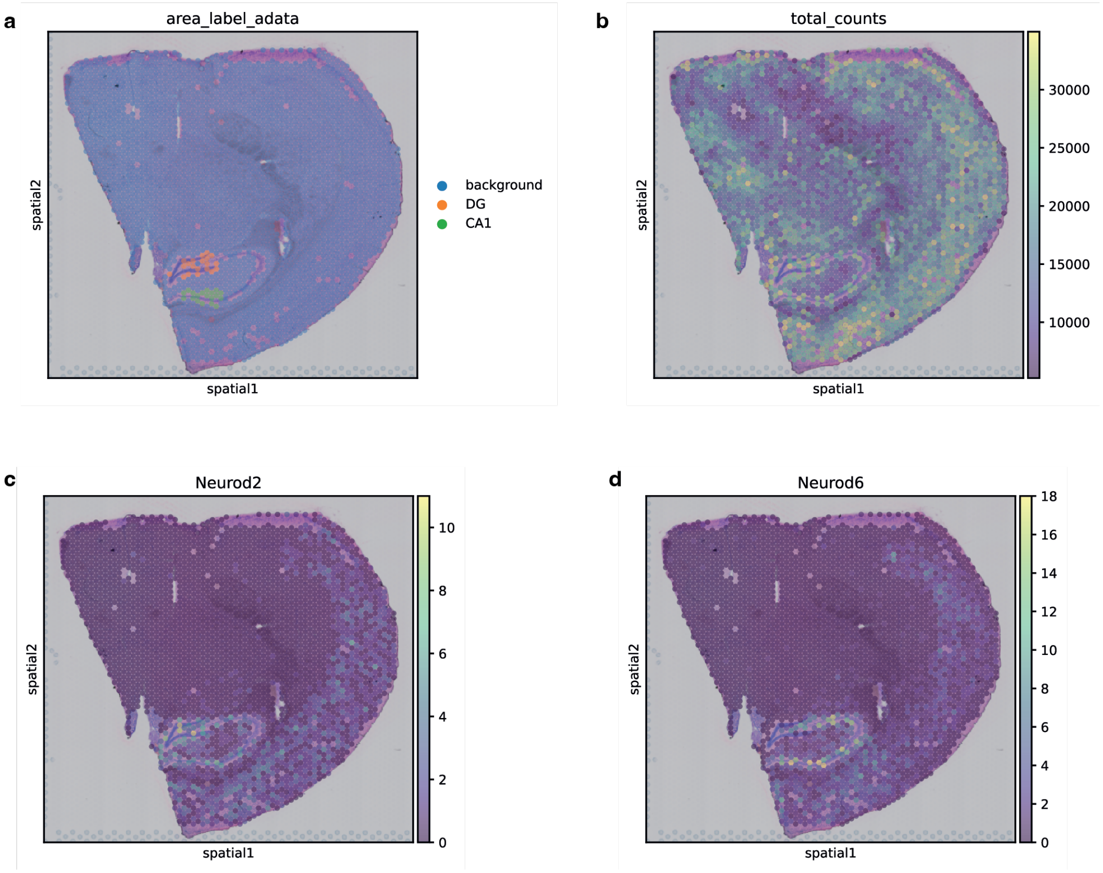


**Supplementary Figure 7. Re-analysis of comparable 10x Visium dataset from adult brain (hippocampus).** H&E image overlayed with the location of the 10x Visium barcode areas[47]. Spots are color-coded to illustrate a) the areas that have been pseudo-bulked as DG and CA1 in our analysis, b) UMIs/spot, c) expression levels for Neurod2 and d) expression levels for Neurod6

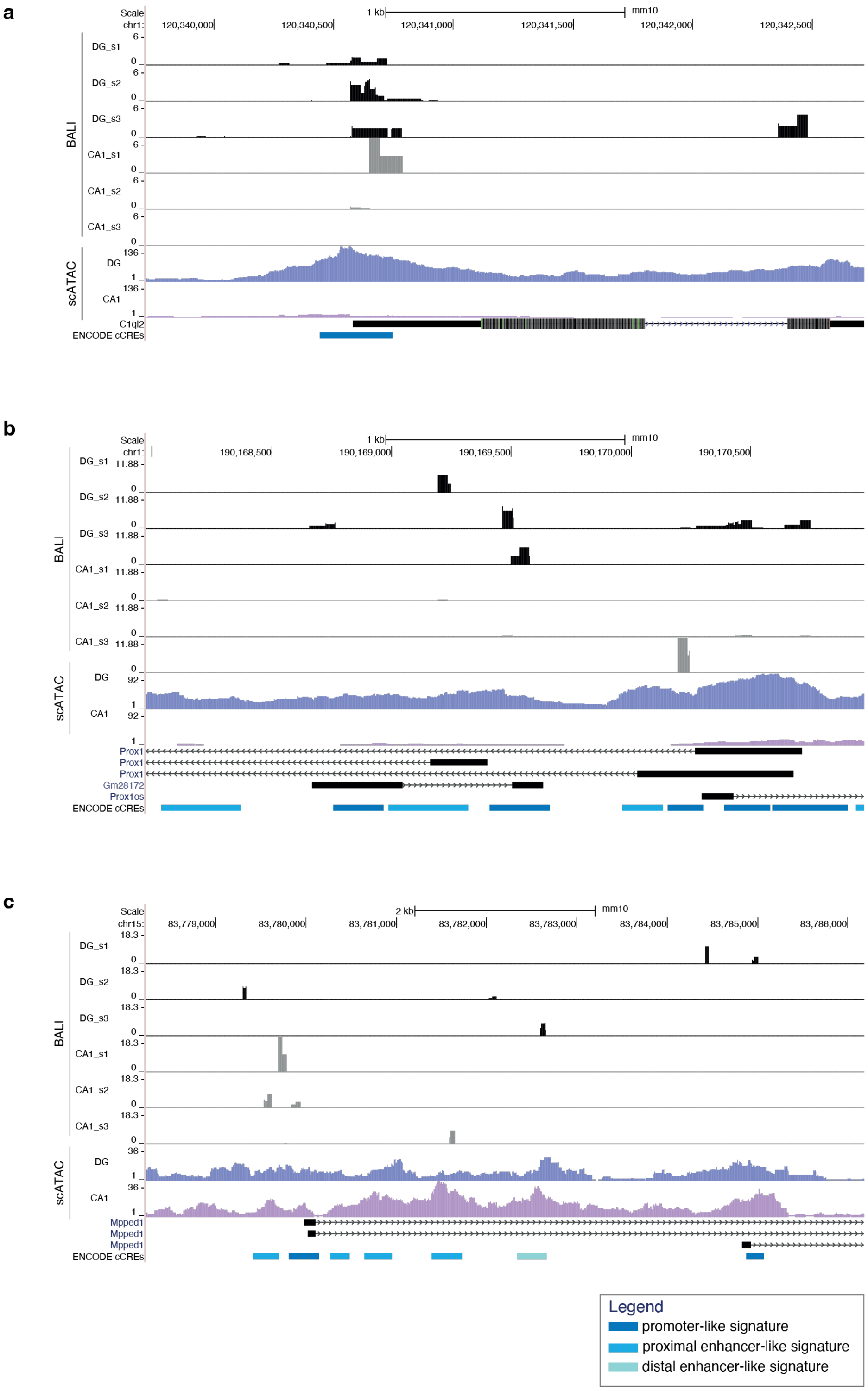
a
Scale
1 kb
mm10
chr1:
120,340,000
120,340,500
120,341,000
120,341,500
120,342,000
120,342,500
BALI
DG_s1
DG_s2
DG_s3
CA1_s1
CA1_s2
CA1_s3
scATAC
DG
CA1
C1ql2
ENCODE cCREs
b
Scale
1 kb
mm10
chr1:
190,168,500
190,169,000
190,169,500
190,170,000
190,170,500
BALI
DG_s1
DG_s2
DG_s3
CA1_s1
CA1_s2
CA1_s3
scATAC
DG
CA1
Prox1
Prox1
Prox1
Gm28172
Prox1os
ENCODE cCREs
c
Scale
2 kb
mm10
chr15:
83,779,000
83,780,000
83,781,000
83,782,000
83,783,000
83,784,000
83,785,000
83,786,000
BALI
DG_s1
DG_s2
DG_s3
CA1_s1
CA1_s2
CA1_s3
scATAC
DG
CA1
Mpped1
Mpped1
Mpped1
ENCODE cCREs
Legend
promoter-like signature
proximal enhancer-like signature
distal enhancer-like signature

**Supplementary Figure 8. Coverage plots for multiomics profiling at additional TSSs.** Accessibility for the DG and CA1 are shown for all replicates separately, and for pseudo-bulked disaggregated scATAC from a comparable sample[47]. For BALI, the DG and CA1 are shown in black and grey, respectively. For sc-ATAC, the DG and CA1 are shown in purple and pink, respectively. The RefSeq annotation is shown below, as well the annotation for regulatory elements from the ENCODE's cCREs database[47] a) C1ql2, b) Prox1, c) Mpped1

| DG exclusive | | | CA1 exclusive | | | shared DG & CA1 | | |
|---|---|---|---|---|---|---|---|---|
| pathway | TF | motif | Pathway | TF | motif | Pathway | TF | motif |
| Neurogenesis | NeuroD1 (q<0.0001) | 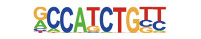 | GABA interneuron | Dlx1 (q<0.0001) | 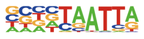 | Chromatin Architecture | Ctcf (q<0.0001) | 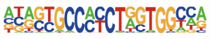 |
| Neurogenesis | NeuroG2 (q<0.0001) | 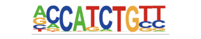 | GABA interneuron | Dlx2 (q<0.0001) | 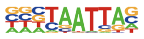 | Chromatin Architecture | Boris (q<0.0001) | 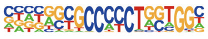 |
| Neurogenesis | E2FA (q<0.0001) | 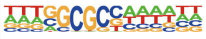 | Regional Specification | Lhx2 (q=0.0001) | 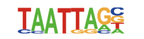 | Chromatin Architecture | YY1 (q<0.0001) | 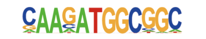 |
| Neurogenesis | Sox15 (q=0.0406) | 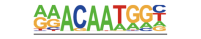 | Regional Specification | Gsx2 (q=0.0003) | 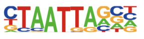 | Constitutive Transcrip-tion | Sp1 (q<0.0001) | 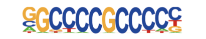 |
| Neurogenesis | Sox17 (q=0.0007) | 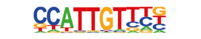 | Regional Specification | Nkx6.1 (q=0.0036) | 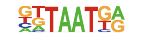 | Constitutive Transcrip-tion | Sp5 (q<0.0001) | 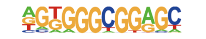 |
| Wnt signalling | Tcf4 (q=0.0039) | 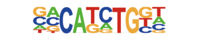 | Neuronal specification | Brn1 (q=0.0005) | 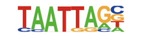 | Constitutive Transcrip-tion | Nfy (q<0.0001) | 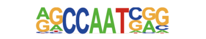 |
| Shh signalling | Gli2 (q<0.0001) | 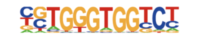 | Neuronal specification | Pbx1 (q=0.0038) | 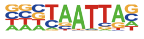 | Neuronal | Rfx1 (q<0.0001) | 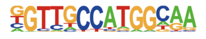 |
| Shh signalling | Gli3 (q<0.0001) | 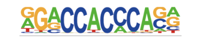 | Plasticity, Memory | Egr1 (q=0.0028) | 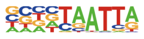 | Neuronal | Rfx2 (q<0.0001) | 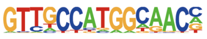 |
| Enhanced Plasticity | Nfat (q<0.0001) | 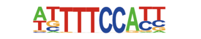 | | | | Neuronal | Olig2 (q=0.0001) | 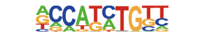 |
| Enhanced Plasticity | Mef2a (q<0.0001) | 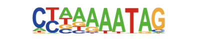 | | | | Neuronal | Atoh1 (q<0.0001) | 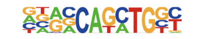 |
| Enhanced Plasticity | Fosl2 (q<0.0001) | 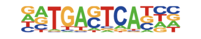 | | | | Neuronal | Atoh7 (q<0.0001) | 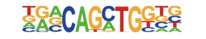 |
| Enhanced Plasticity | Atf4 (q=0.0001) | 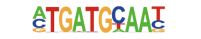 | | | | Activity-dependent Plasticity | Fos (q<0.0001) | 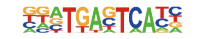 |
| Metabolic Adaptation | Tfe3 (q=0.0006) | 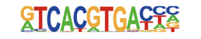 | | | | Activity-dependent Plasticity | Jun (q<0.0001) | 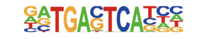 |
| Metabolic Adaptation | Foxo1 (q=0.01) | 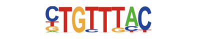 | | | | Activity-dependent Plasticity | AP-1 (q=0.0001) | 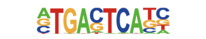 |
| Stem Cell | Oct4 (q=0.0024) | 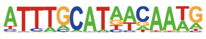 | | | | Memory | CEBP (q<0.0001) | 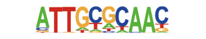 |

**Supplementary Figure 9. DNA motif enrichment in BALI multi-omic data.** List of transcription factors and DNA binding molecules which motifs have been detected by HOMER analysis as enriched in BALI ATAC peaks vs. background genome regions. Factors specific for the dentate gyrus region are on the left, factors specific for the CA1 region are in the center, and factors enriched in both regions are on the right. For each factor we report the general function or relevant biological pathway, name, q-value, and logo.

**Table 1**

| Parameter | Value | Notes |
|---|---|---|
| Flow cell volume | ~280ul | 20mm diameter x 0.8mm thickness |
| Reagent volume required per cycle | ~300ul | |
| Dead volume | nil | Valveless design, bespoke pipetting robot is used to transfer fluid |
| Pipetting arm positioning precision | 0.1mm | |
| Uncaging resolution | 2um / pixel | |
| Uncaging wavelength | 405nm | |
| Uncaging power on image plane | 39W | |
| Uncaging contrast | 1:400 ANSI | |
| Uncaging uniformity | (see Fig 5c) | |
| Uncaging field of view | 3 x 5 mm | |
| Imaging options | Can be user-configured | • 4x or 10x objective<br>• Adapter for solid-state light engine (i.e. Spectra X or SOLA)<br>• Excitation/emission filters for common fluorophores (DAPI, GFP/FITC, Tritc, Texas Red, cy5) |
| Time required per ligation cycle | 90 min | assuming "standard protocol": 2 min uncaging, 45 min ligation, 10 min + 5 min(x4) washes |
| Time for full run (1024 areas) | 33h | Including initial ligation to install photocage and final ligation to install sequencing adapters |
| Sample size | 3.14 $cm^2$ | only limited by flow cell – other designs possible |
| Rough cost of parts | 35,000 GBP | Most important component is the DLP projector (20-25k GBP). Full parts list available at https://github.com/giorgiabattistoni/BALI. |

**Table 2**
NB: Unless noted, all oligonucleotides were purchased from Integrated DNA Technologies (IDT)

| Oligo name | Sequence | Notes |
|---|---|---|
| BL354 | AATGATACGGCGACCACCGAGATCTACACTC TTTCCCTACACGACGCTCTTCCGATCT | overhang screen |
| BL355 | /5Phos/NNNNNN AGATCGGAAGAGCGTCGTGTAGGGAAAGAGTGTA GATCTCGGTGGTCGCCGTATCATT | overhang screen |
| BL356 | /5Phos/NNNNNN AGATCGGAAGAGCACACGTCTGAA CTCCAGTCAC GCCAAT ATCTCGTATGCCGTCTTCTGCTTG | overhang screen |
| BL357 | CAAGCAGAAGACGGCATACGAGAT ATTGGC GTGACTGGAGTTCAGACGTGTGCTCTTCCGATCT | overhang screen |
| BL354b | AATGATACGGCGACCACCGAGATCTACAC TCTTTCCCTACACGACGCTCTTCCGATCT | overhang screen |
| BL355b | /5Phos/NNNNNNN AGATCGGAAGAGCGTCGTGTAGGGAAAGAGTG TAGATCTCGGTGGTCGCCGTATCATT | overhang screen |
| BL356b | /5Phos/NNNNNNN AGATCGGAAGAGCACACGTCT GAACTCCAGTCAC CTTGTA ATCTCGTATGCCGTCTTCTGCTTG | overhang screen |
| BL357b | CAAGCAGAAGACGGCATACGAGAT TACAAG GTGACTGGAGTTCAGACGTGTGCTCTTCCGATCT | overhang screen |
| BL092_50nt | /5Phos/GTGCGT ATGTCGAGAGCTAGCCGCGGAATTCTATACCAA TTAGAGTCTCC/3Cy5Sp/ | fluorescent root oligo |
| BL407 | /5Phos/GGTATTCGACGAGTCCGC | OH screen validation |
| BL408 | /5Phos/GGTTATCGACGAGTCCGC | OH screen validation |
| BL409 | /5Phos/GGTAATCGACGAGTCCGC | OH screen validation |
| BL410 | /5Phos/ATAACCCGACGAGTCCGC | OH screen validation |
| BL411 | /5Phos/GGTTTACGACGAGTCCGC | OH screen validation |
| BL412 | /5Phos/AATACCCGACGAGTCCGC | OH screen validation |
| BL413 | /5Phos/ATTACCCGACGAGTCCGC | OH screen validation |
| BL414 | /5Phos/GGATTTCGACGAGTCCGC | OH screen validation |
| BL415 | /5Phos/GTTGTTCGACGAGTCCGC | OH screen validation |
| BL416 | /5Phos/GGGTTTCGACGAGTCCGC | OH screen validation |
| BL417 | /5Phos/GCATGCCGACGAGTCCGC | OH screen validation |
| BL418 | /5Phos/CGCGAGCGACGAGTCCGC | OH screen validation |
| BL419 | /5Phos/GCTAGCCGACGAGTCCGC | OH screen validation |
| BL420 | /5Phos/CTCGCGCGACGAGTCCGC | OH screen validation |
| BL421 | /5Phos/CTGCAGCGACGAGTCCGC | OH screen validation |
| BL422 | /5Phos/GCGTGCCGACGAGTCCGC | OH screen validation |
| BL423 | /5Phos/GCACGCCGACGAGTCCGC | OH screen validation |
| BL424 | /5Phos/GCTCGCCGACGAGTCCGC | OH screen validation |
| BL425 | /5Phos/GCGAGCCGACGAGTCCGC | OH screen validation |
| BL426 | /5Phos/GCGCGCCGACGAGTCCGC | OH screen validation |
| BL452 | /5Phos/AGATCGGCGACGAGTCCGC | OH screen validation |
| BL453 | /5Phos/AATAACCCGACGAGTCCGC | OH screen validation |
| BL454 | /5Phos/ATTAACCCGACGAGTCCGC | OH screen validation |
| BL455 | /5Phos/ATAAACCCGACGAGTCCGC | OH screen validation |
| BL456 | /5Phos/GGTTATTCGACGAGTCCGC | OH screen validation |
| BL457 | /5Phos/GGTATATCGACGAGTCCGC | OH screen validation |
| BL458 | /5Phos/GGTAATTCGACGAGTCCGC | OH screen validation |
| BL459 | /5Phos/AAACCATCGACGAGTCCGC | OH screen validation |
| BL460 | /5Phos/AATTACCCGACGAGTCCGC | OH screen validation |
| BL461 | /5Phos/AAATACCCGACGAGTCCGC | OH screen validation |
| BL462 | /5Phos/GCATCGCCGACGAGTCCGC | OH screen validation |
| BL463 | /5Phos/GCGATGCCGACGAGTCCGC | OH screen validation |
| BL464 | /5Phos/GCTCTGCCGACGAGTCCGC | OH screen validation |

| | | |
|---|---|---|
| BL465 | /5Phos/GCGTCGCCGACGAGTCCGC | OH screen validation |
| BL466 | /5Phos/GCGTAGCCGACGAGTCCGC | OH screen validation |
| BL467 | /5Phos/GCAGAGCCGACGAGTCCGC | OH screen validation |
| BL468 | /5Phos/GCTACGCCGACGAGTCCGC | OH screen validation |
| BL469 | /5Phos/CCTACGGCGACGAGTCCGC | OH screen validation |
| BL470 | /5Phos/CCTGCGGCGACGAGTCCGC | OH screen validation |
| BL471 | /5Phos/GCGAAGCCGACGAGTCCGC | OH screen validation |
| BL427 | ACGCACGCGGACTCGTCG | OH screen validation |
| BL428 | AATACCGCCCATACGTCG | OH screen validation |
| BL429 | ATAACCGCCCATACGTCG | OH screen validation |
| BL430 | ATTACCGCCCATACGTCG | OH screen validation |
| BL431 | GGTTATGCCCATACGTCG | OH screen validation |
| BL432 | TAAACCGCCCATACGTCG | OH screen validation |
| BL433 | GGTATTGCCCATACGTCG | OH screen validation |
| BL434 | GGTAATGCCCATACGTCG | OH screen validation |
| BL435 | AAATCCGCCCATACGTCG | OH screen validation |
| BL436 | AACAACGCCCATACGTCG | OH screen validation |
| BL437 | AAACCCGCCCATACGTCG | OH screen validation |
| BL438 | GCATGCGCCCATACGTCG | OH screen validation |
| BL439 | CTCGCGGCCCATACGTCG | OH screen validation |
| BL440 | GCTAGCGCCCATACGTCG | OH screen validation |
| BL441 | CGCGAGGCCCATACGTCG | OH screen validation |
| BL442 | CTGCAGGCCCATACGTCG | OH screen validation |
| BL443 | GCACGCGCCCATACGTCG | OH screen validation |
| BL444 | GCGTGCGCCCATACGTCG | OH screen validation |
| BL445 | GCGAGCGCCCATACGTCG | OH screen validation |
| BL446 | GCTCGCGCCCATACGTCG | OH screen validation |
| BL447 | GCGCGCGCCCATACGTCG | OH screen validation |
| BL472 | CCGATCTGCCCATACGTCG | OH screen validation |
| BL473 | GGTTATTGCCCATACGTCG | OH screen validation |
| BL473b | GGTTAATGCCCATACGTCG | OH screen validation |
| BL474 | GGTTTATGCCCATACGTCG | OH screen validation |
| BL474b | AATAACCGCCCATACGTCG | OH screen validation |
| BL475 | ATATACCGCCCATACGTCG | OH screen validation |
| BL475b | AATTACCGCCCATACGTCG | OH screen validation |
| BL476 | ATGGTTTGCCCATACGTCG | OH screen validation |
| BL476b | GGTAATTGCCCATACGTCG | OH screen validation |
| BL477 | GGTATTTGCCCATACGTCG | OH screen validation |
| BL478 | GCGATGCGCCCATACGTCG | OH screen validation |
| BL479 | GCATCGCGCCCATACGTCG | OH screen validation |
| BL480 | GCAGAGCGCCCATACGTCG | OH screen validation |
| BL481 | GCGACGCGCCCATACGTCG | OH screen validation |
| BL482 | GCTACGCGCCCATACGTCG | OH screen validation |
| BL483 | GCTCTGCGCCCATACGTCG | OH screen validation |
| BL484 | GCGTAGCGCCCATACGTCG | OH screen validation |
| BL485 | CCGTAGGGCCCATACGTCG | OH screen validation |
| BL486 | CCGCAGGGCCCATACGTCG | OH screen validation |
| BL487 | GCTTCGCGCCCATACGTCG | OH screen validation |
| BL448 | GTGCGTCGACGTATGGGC | OH screen validation |
| polyT-rootV2 | [NH2] TTTTTTTTTTTTTTTTTTTTTTTTTTTTTTTTGCCTAA TACGACTCACTATAGGGAGACTCTAATTGGTATAGA ATTCCGCGGCTAGCTCTCGACAT | anchor oligo for tissue |
| BL940 | /5ATTO488N/TAA/iSpPC/GGTATT ATGTCGAGAG CAATTAGAGT CGCGCCTTAAGATAC AGATCGGAAGAGCGTCGTGTAG CGCGCCTTAAGATAC/3BioTEG/ | biotin root oligo |
| BL003 | [Cy3] [PC spacer] CTAGGTCG ATGTCGAGAG CAATTAGAGT CGCGCCTTAAGATAC | amino root oligo (NB: from biomers) |

| | | |
|---|---|---|
| | AGATCGGAAGAGCGTCGTGTAG [int. Thiol C6 SS] [Aminolink C6] | |
| BL728 | /5Cy3/TAA/iSpPC/GGTATT ATGTCGAGAG CAATTAGAGT CGCGCCTTAAGATAC AGATCGGAAGAGCGTCGTGTAG CGCGCCTTAAGATAC/3AmMC6T/ | amino root oligo |
| lig_test_onbridge1_fw | /5Cy5/GGTAGTAT AGCTACCATG | first ligation experiment |
| lig_test_onbridge1_rev | CGACCTAG CATGGTAGCT | first ligation experiment |
| BL641 | AATACC CG GGACTCGT GC | fluorescent indices |
| BL642 | AATACC CG ATCTAGGT GC | fluorescent indices |
| BL643 | GGTTATT CG ATCTAGGT GC | fluorescent indices |
| BL644 | GGTTATT CG GGACTCGT GC | fluorescent indices |
| BL645 | /5ATTO565N/AT/iSpPC/ AATAACC GC ACGAGTCC CG | fluorescent indices |
| BL646 | /5ACy5AT/iSpPC/ AATAACC GC ACCTAGAT CG | fluorescent indices |
| BL647 | /5Cy5/AT/iSpPC/ GGTATT GC ACCTAGAT CG | fluorescent indices |
| BL648 | /5ATTO565N/AT/iSpPC/ GGTATT GC ACGAGTCC CG | fluorescent indices |
| BL736 | /56-FAM/AT/iSpPC/ AATAACC GC ACGAGTCC CG | fluorescent indices |
| BL737 | /56-FAM/AT/iSpPC/ GGTATT GC ACGAGTCC CG | fluorescent indices |
| BL092 | /5Phos/ GTGCGT ATGTCGAGAGCTAGC CGCGGAATTCTATAC CAATTAGAGT CTCCCTATAGTGAGTCGTATTA GGC [cy5] | fluorescent root oligo |
| BL45 | ACGCACCGGGACTCGTGC | ligation on beads |
| BL46 | /5Phos/GTGGACCGCACGAGTCCCG | ligation on beads |
| BL47 | /5Phos/GTGCGTGCACGTATGGCG | ligation on beads |
| BL48 | GGTCCACCGCCATACGTGC | ligation on beads |
| poly-T root | /5Phos/GTGCGTATGTCGAGAGCAATTAGAGTNNNNNNNNNAGATCGGAAGAGCGTCGTGTAGCTCCCTATAGTGAGTCGTATTACTAGCTAGCGTTTTTTTTTTTTTTTTTTTTTTTTTTTTT/3AmMO/ | anchor oligo for tissue |
| BL603 | /5Phos/ GGTATT GC ACCTAGAT CG | ligation on tissue |
| BL604 | /5Phos/ AATAACC GC ACGAGTCC CG | ligation on tissue |
| BL611 | /5Phos/ GGTATT ATGTCGAGAGCTAGCCGCGGAATTCTATACCA ATTAGAGTCTCC/3Cy5Sp/ | fluorescent root for tissue |
| BL583 | GGTTATT CG ATCTAGGT GC | ligation on tissue |
| BL585 | AATACC CG GGACTCGT GC | ligation on tissue |
| BL621 | TAA/iSpPC/AATAACC CTAG NNNNNNNN ACTG TTTTTTTTTTTTTTTTTTTTTTTTTTTTTTVN | caged RT primer |
| BL599 | GGTTATT CG ATCTAGGT GC | BALI-RT |
| BL623 | /5deSBioTEG/AGACGTGTGCTCTTCCGATCT NNNNNN GC ACCTAGAT CG | BALI-RT |
| BL601 | GGTTATT CG GGACTCGT GC | BALI-RT |
| BL624 | /5deSBioTEG/AGACGTGTGCTCTTCCGATCT NNNNNN GC ACGAGTCC CG | BALI-RT |
| BL617 | tacactctttccctacacgacgctcttccgatct rGrGrG | TSO oligo |
| universal truseq primer | AATGATACGGCGACCACCGAGATCTACAC TCTTTCCCTACACGACGCTCTTCCGATCT | Illumina library preparation |
| A001 TS index primer | CAAGCAGAAGACGGCATACGAGAT CGTGAT GTGACTGGAGTTCAGACGTGTGCTCTTCCGATC | Illumina library preparation |
| A002 TS index primer | CAAGCAGAAGACGGCATACGAGAT acatcg GTGACTGGAGTTCAGACGTGTGCTCTTCCGATC | Illumina library preparation |
| A003 TS index primer | CAAGCAGAAGACGGCATACGAGAT gcctaa GTGACTGGAGTTCAGACGTGTGCTCTTCCGATC | Illumina library preparation |
| A004 TS index primer | CAAGCAGAAGACGGCATACGAGAT tggtca GTGACTGGAGTTCAGACGTGTGCTCTTCCGATC | Illumina library preparation |
| A005 TS index primer | CAAGCAGAAGACGGCATACGAGAT cactgt GTGACTGGAGTTCAGACGTGTGCTCTTCCGATC | Illumina library preparation |
| A006 TS index primer | CAAGCAGAAGACGGCATACGAGAT attggc GTGACTGGAGTTCAGACGTGTGCTCTTCCGATC | Illumina library preparation |

| | | |
|---|---|---|
| A007 TS index primer | CAAGCAGAAGACGGCATACGAGAT gatctg GTGACTGGAGTTCAGACGTGTGCTCTTCCGATC | Illumina library preparation |
| A008 TS index primer | CAAGCAGAAGACGGCATACGAGAT tcaagt GTGACTGGAGTTCAGACGTGTGCTCTTCCGATC | Illumina library preparation |
| A009 TS index primer | CAAGCAGAAGACGGCATACGAGAT ctgatc GTGACTGGAGTTCAGACGTGTGCTCTTCCGATC | Illumina library preparation |
| A010 TS index primer | CAAGCAGAAGACGGCATACGAGAT aagcta GTGACTGGAGTTCAGACGTGTGCTCTTCCGATC | Illumina library preparation |
| A011 TS index primer | CAAGCAGAAGACGGCATACGAGAT gtagcc GTGACTGGAGTTCAGACGTGTGCTCTTCCGATC | Illumina library preparation |
| A012 TS index primer | CAAGCAGAAGACGGCATACGAGATtacaagGTGA CTGGAGTTCAGACGTGTGCTCTTCCGATC | Illumina library preparation |
| BL699 | /5Phos/GGTATTATGGAACGTGATAGATGT GTATAAGAGACAG | BALI tn5 adapter |
| BL538 | /5Phos/CTGTCTCTTATACACATCT GATCGACT /3InvdT/ | rev tn5 adapter |
| BL515 | TCGTCGGCAGCGTCAGATGTGTATAAGAGACAG | standard tn5 A adapter |
| BL749 | /5BiotinTEG/TCGTCGGCAGCGTCAGATGTGTA TAAGAGACAG | standard tn5 A adapter, multi-omic |
| BL680 | AAT/iSpPC/ AATAACC GC ACTCAGGT CG | BALI-ATAC indices |
| BL676 | AATACC CG ACCTGAGT GC | BALI-ATAC indices |
| BL681 | AAT/iSpPC/ GGTATT GC ACGGACGA CG | BALI-ATAC indices |
| BL677 | GGTTATT CG TCGTCCGT GC | BALI-ATAC indices |
| BL683 | AAT/iSpPC/ AATAACC GC ACCTAGAT CG | BALI-ATAC indices |
| BL642 | AATACC CG ATCTAGGT GC | BALI-ATAC indices |
| BL732 | AGACGTGTGCTCTTCCGATCT NNNNNN GC ACGAGTCC CG | BALI-ATAC indices |
| BL601 | GGTTATT CG GGACTCGT GC | BALI-ATAC indices |
| BL733 | AGACGTGTGCTCTTCCGATCT NNNNNN GC ACGGACGA CG | BALI-ATAC indices |
| BL677 | GGTTATT CG TCGTCCGT GC | BALI-ATAC indices |
| BL732 | AGACGTGTGCTCTTCCGATCT NNNNNN GC ACGAGTCC CG | BALI-ATAC indices |
| BL641 | AATACC CG GGACTCGT GC | BALI-ATAC indices |
| BL727 | AATGATACGGCGACCACCGAGATCTACACTC GTCGGCAGCGTCAG | BALI-ATAC library prep |
| BL730 | /5Phos/GGTATT CTAGACTG TTTTTTTTTTTTTTTTTTTTTTTTTTTTTTVN | RT primer |
| BL752 | CCTACACGACGCTCTTCCG | RNA library prep |
| BL753 | AGACGTGTGCTCTTCCGATC | RNA library prep |
| BL371 | /5Phos/CTGTCTCTTATACACATCT | tn5 adapter for cDNA |
| Ad2.1_TAAGGCGA | CAAGCAGAAGAC GGC ATA CGA GAT TCG CCT TAG TCT CGT GGG CTC GGA GAT GT | BALI-ATAC library prep |
| Ad2.2_CGTACTAG | CAA GCA GAA GAC GGC ATA CGA GAT CTA GTA CGG TCT CGT GGG CTC GGA GAT GT | BALI-ATAC library prep |
| Ad2.3_AGGCAGAA | CAA GCA GAA GAC GGC ATA CGA GAT TTC TGC CTG TCT CGT GGG CTC GGA GAT GT | BALI-ATAC library prep |
| Ad2.4_TCCTGAGC | CAA GCA GAA GAC GGC ATA CGA GAT GCT CAG GAG TCT CGT GGG CTC GGA GAT GT | BALI-ATAC library prep |
| Ad2.5_GGACTCCT | CAA GCA GAA GAC GGC ATA CGA GAT AGG AGT CCG TCT CGT GGG CTC GGA GAT GT | BALI-ATAC library prep |
| Ad2.6_TAGGCATG | CAA GCA GAA GAC GGC ATA CGA GAT CAT GCC TAG TCT CGT GGG CTC GGA GAT GT | BALI-ATAC library prep |
| Ad2.7_CTCTCTAC | CAA GCA GAA GAC GGC ATA CGA GAT GTA GAG AGG TCT CGT GGG CTC GGA GAT GT | BALI-ATAC library prep |
| Ad2.8_CAGAGAGG | CAA GCA GAA GAC GGC ATA CGA GAT CCT CTC TGG TCT CGT GGG CTC GGA GAT GT | BALI-ATAC library prep |
| Ad2.9_GCTACGCT | CAA GCA GAA GAC GGC ATA CGA GAT AGC GTA GCG TCT CGT GGG CTC GGA GAT GT | BALI-ATAC library prep |

| | | |
|---|---|---|
| Ad2.10_CGAGGCTG | CAA GCA GAA GAC GGC ATA CGA GAT CAG CCT CGG TCT CGT GGG CTC GGA GAT GT | BALI-ATAC library prep |
| Ad2.11_AAGAGGCA | CAA GCA GAA GAC GGC ATA CGA GAT TGC CTC TTG TCT CGT GGG CTC GGA GAT GT | BALI-ATAC library prep |
| Ad2.12_GTAGAGGA | CAA GCA GAA GAC GGC ATA CGA GAT TCC TCT ACG TCT CGT GGG CTC GGA GAT GT | BALI-ATAC library prep |

**Supplementary movie 1 legend**

Video outlining the BALI combinatorial ligation process, in which the step-wise addition of DNA indices to a growing chain (triggered by light-activation) results in the production of a complex multi-unit spatial barcode

**Supplementary movie 2 legend**

Combinatorial barcode writing for 16 areas (2 indices / 4 cycles - main text Fig. 1c). The video was generated by taking a multichannel (FITC / cy5) confocal microscope image after the ligation of the final index for each cycle. Brightness/contrast for each image were adjusted to make the signal intensity consistent. Images are in pseudo-colors in a color-blind friendly palette

**Supplementary movie 3 legend**

Combinatorial barcode writing for 256 areas (2 indices / 8 cycles – main text Fig. 5). The video was generated by taking a multichannel (ATTO 565 / cy5) confocal microscope image after the ligation of the final index for each cycle. Brightness/contrast for each image were adjusted to make the signal intensity consistent. Images are in pseudo-colors in a color-blind friendly palette

PNAS

## IMAXT Cancer Grand Challenge Consortium Authors

The following authors were part of the IMAXT Cancer Grand Challenge Consortium:

Bruno Albuquerque (1), Martina Alini (1), Heather Ashmore (1), Thomas Ashmore (1), Giorgia Battistoni (1), Dario Bressan (1), Ian Gordon Cannell (1), Hannah Casbolt (1), Lauren Deighton (1), Ilaria Falciatori (1), Carla Boquetale (1), Nikki Coutts (1), Chee Ying Sia (1), Atefeh Fatemi (1), Nicole Hemmer (1), Kui Hua (1), Cristina Jauset (1), Tatjana Kovačević (1), Claire M Mulvey (1), Natasha Narayanan (1), Fiona Nugent (1), Clare Rebbeck (1), Marta Paez Ribes (1), Isabella Pearsall (1), Sarah Pearsall (1), Fatime Qosaj (1), Kirsty Sawicka (1), Sophia A Wild (1), Elena Williams (1), Hamid Raza Ali (1), Samuel Aparicio (1), Emma Laks (2), Yangguang Li (2), Ciara H O'Flanagan (2), Austin Smith (2), Teresa Ruiz (2), Daniel Lai (2), Andrew Roth (2), Vinci Au (2), Caroline Baril (2), Sean Beatty (2), Shankar Balasubramanian (2), João CF Nogueira (1, Max Lee (1, Bernd Bodenmiller (), Alina Bollhagen (4), Marcel Burger (4), Laura Kuett (4), Jonas Windhager (4), Edward S Boyden (4), Debarati Ghosh (5), Anubhav Sinha (5), Brett Pryor (5), Ruihan Zhang (5), Jack Lovell (5), Chi Zhang (5), Yangning Lu (5), Carlos Caldas (5), Alejandra Bruna (1), Maurizio Callari (1), Lauren Deighton (1), Wendy Greenwood (1), Giulia Lerda (1), Yaniv Eyal-Lubling (1), Oscar M Rueda (1), Abigail Shea (1), Owen Harris (1), Robby Becker (6), Natalie Duncan (6), Flaminia Grimaldi (6), Suvi Harris (6), Sara Lisa Vogl (6), Joanna Weselak (6), Johanna A Joyce (6), Spencer S Watson (7), John Marioni (7), Sohrab P Shah (1), Andrew McPherson (2, Ignacio Vázquez-García (2, Simon Tavaré (8), Khanh N Dinh (1, Russell Kunes (9), Nicholas A Walton (9), Mohammad Al Sa'd (10), Nick Chornay (10), Ali Dariush (10), Eduardo A González-Solares (10), Carlos González-Fernández (10), Melis Irfan (10), Aybüke Küpcü Yoldaş (10), Alireza Molaeinezhad (10), Neil Millar (10), Leigh Smith (10), Tristan Whitmarsh (10), Xiaowei Zhuang (10), Jean Fan (11), Hsuan Lee (11), Leonardo A Sepúlveda (11), Chenglong Xia (11), Pu Zheng (11)

(1) Cancer Research UK Cambridge Institute, Li Ka Shing Centre, University of Cambridge, Cambridge CB2 0RE, UK
(2) Department of Molecular Oncology, BC Cancer, part of the Provincial Health Services Authority, Vancouver, BC, Canada
(3) Department of Chemistry, University of Cambridge, Lensfield Road, Cambridge, CB2 1EW, UK
(4) Department of Quantitative Biomedicine, University of Zurich, Zurich 8057, Switzerland
(5) McGovern Institute, Departments of Biological Engineering and Brainand Cognitive Sciences, Massachusetts Institute of Technology,Cambridge, Massachusetts, USA, and HHMI, Cambridge, Massachusetts, USA
(6) Súil Interactive Ltd, Dame Lane, Dublin, UK
(7) Department of Oncology and Ludwig Institute for Cancer Research, University of Lausanne, Lausanne, Switzerland
(8) Computational Oncology, Department of Epidemiology and Biostatistics, Memorial Sloan Kettering Cancer Center, New York, USA
(9) Herbert and Florence Irving Institute for Cancer Dynamics, Columbia University, New York, NY, USA
(10) Institute of Astronomy, University of Cambridge, Madingley Road, Cambridge, CB3 0HA, UK
(11) Howard Hughes Medical Institute, Harvard University, Cambridge, MA 02138, USA; Department of Physics, Harvard University, Cambridge, MA 02138, USA; Department of Chemistry and Chemical Biology, Harvard University, Cambridge, MA 02138, USA
