# Supplementary material for: Spatially tunable multiomic sequencing using light-driven combinatorial barcoding of molecules in tissues
Source: Proc Natl Acad Sci U S A. 2026 May 18;123(21):e2527896123. doi: 10.1073/pnas.2527896123 (PMC13214022; doi:10.1073/pnas.2527896123)
Supplement: Supplementary file 2 — Dataset S01 (DOCX) [file pnas.2527896123.sd01.docx]

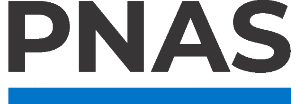


**IMAXT Cancer Grand Challenge Consortium Authors**

The following authors were part of the IMAXT Cancer Grand Challenge Consortium:

Bruno Albuquerque (1), Martina Alini (1), Heather Ashmore (1), Thomas Ashmore (1), Giorgia Battistoni (1), Dario Bressan (1), Ian Gordon Cannell (1), Hannah Casbolt (1), Lauren Deighton (1), Ilaria Falciatori (1), Carla Boquetale (1), Nikki Coutts (1), Chee Ying Sia (1), Atefeh Fatemi (1), Nicole Hemmer (1), Kui Hua (1), Cristina Jauset (1), Tatjana Kovačević (1), Claire M Mulvey (1), Natasha Narayanan (1), Fiona Nugent (1), Clare Rebbeck (1), Marta Paez Ribes (1), Isabella Pearsall (1), Sarah Pearsall (1), Fatime Qosaj (1), Kirsty Sawicka (1), Sophia A Wild (1), Elena Williams (1), Hamid Raza Ali (1), Samuel Aparicio (1), Emma Laks (2), Yangguang Li (2), Ciara H O'Flanagan (2), Austin Smith (2), Teresa Ruiz (2), Daniel Lai (2), Andrew Roth (2), Vinci Au (2), Caroline Baril (2), Sean Beatty (2), Shankar Balasubramanian (2), João CF Nogueira (1, Max Lee (1, Bernd Bodenmiller (), Alina Bollhagen (4), Marcel Burger (4), Laura Kuett (4), Jonas Windhager (4), Edward S Boyden (4), Debarati Ghosh (5), Anubhav Sinha (5), Brett Pryor (5), Ruihan Zhang (5), Jack Lovell (5), Chi Zhang (5), Yangning Lu (5), Carlos Caldas (5), Alejandra Bruna (1), Maurizio Callari (1), Lauren Deighton (1), Wendy Greenwood (1), Giulia Lerda (1), Yaniv Eyal-Lubling (1), Oscar M Rueda (1), Abigail Shea (1), Owen Harris (1), Robby Becker (6), Natalie Duncan (6), Flaminia Grimaldi (6), Suvi Harris (6), Sara Lisa Vogl (6), Joanna Weselak (6), Johanna A Joyce (6), Spencer S Watson (7), John Marioni (7), Sohrab P Shah (1), Andrew McPherson (2, Ignacio Vázquez-García (2, Simon Tavaré (8), Khanh N Dinh (1, Russell Kunes (9), Nicholas A Walton (9), Mohammad Al Sa'd (10), Nick Chornay (10), Ali Dariush (10), Eduardo A González-Solares (10), Carlos González-Fernández (10), Melis Irfan (10), Aybüke Küpcü Yoldaş (10), Alireza Molaeinezhad (10), Neil Millar (10), Leigh Smith (10), Tristan Whitmarsh (10), Xiaowei Zhuang (10), Jean Fan (11), Hsuan Lee (11), Leonardo A Sepúlveda (11), Chenglong Xia (11), Pu Zheng (11)

(1) Cancer Research UK Cambridge Institute, Li Ka Shing Centre, University of Cambridge, Cambridge CB2 0RE, UK

(2) Department of Molecular Oncology, BC Cancer, part of the Provincial Health Services Authority, Vancouver, BC, Canada

(3) Department of Chemistry, University of Cambridge, Lensfield Road, Cambridge, CB2 1EW, UK

(4) Department of Quantitative Biomedicine, University of Zurich, Zurich 8057, Switzerland

(5) McGovern Institute, Departments of Biological Engineering and Brainand Cognitive Sciences, Massachusetts Institute of Technology,Cambridge, Massachusetts, USA, and HHMI, Cambridge, Massachusetts, USA

(6) Súil Interactive Ltd, Dame Lane, Dublin, UK

(7) Department of Oncology and Ludwig Institute for Cancer Research, University of Lausanne, Lausanne, Switzerland

(8) Computational Oncology, Department of Epidemiology and Biostatistics, Memorial Sloan Kettering Cancer Center, New York, USA

(9) Herbert and Florence Irving Institute for Cancer Dynamics, Columbia University, New York, NY, USA

(10) Institute of Astronomy, University of Cambridge, Madingley Road, Cambridge, CB3 0HA, UK

(11) Howard Hughes Medical Institute, Harvard University, Cambridge, MA 02138, USA; Department of Physics, Harvard University, Cambridge, MA 02138, USA; Department of Chemistry and Chemical Biology, Harvard University, Cambridge, MA 02138, USA
